# Linked-read sequencing identifies abundant microinversions and introgression in the arboviral vector *Aedes aegypti*

**DOI:** 10.1186/s12915-020-0757-y

**Published:** 2020-03-12

**Authors:** Seth N. Redmond, Atashi Sharma, Igor Sharakhov, Zhijian Tu, Maria Sharakhova, Daniel E. Neafsey

**Affiliations:** 1 Institute of Vector Borne Disease, Monash University, Melbourne, Australia; 2 Harvard TH Chan School of Public Health, Boston, MA USA; 3 Fralin Life Science Institute, Virginia Polytechnic and State University, Blacksburg, VA USA; 4 Broad Institute of MIT and Harvard, Cambridge, MA USA

**Keywords:** Structural variation, Chromosomal inversions, Introgression, *Aedes aegypti*

## Abstract

**Background:**

*Aedes aegypti* is the principal mosquito vector of Zika, dengue, and yellow fever viruses. Two subspecies of *Ae. aegypti* exhibit phenotypic divergence with regard to habitat, host preference, and vectorial capacity. Chromosomal inversions have been shown to play a major role in adaptation and speciation in dipteran insects and would be of great utility for studies of *Ae. aegypti.* However, the large and highly repetitive genome of *Ae. aegypti* makes it difficult to detect inversions with paired-end short-read sequencing data, and polytene chromosome analysis does not provide sufficient resolution to detect chromosome banding patterns indicative of inversions.

**Results:**

To characterize chromosomal diversity in this species, we have carried out deep Illumina sequencing of linked-read (10X Genomics) libraries in order to discover inversion loci as well as SNPs. We analyzed individuals from colonies representing the geographic limits of each subspecies, one contact zone between subspecies, and a closely related sister species. Despite genome-wide SNP divergence and abundant microinversions, we do not find any inversions occurring as fixed differences between subspecies. Many microinversions are found in regions that have introgressed and have captured genes that could impact behavior, such as a cluster of odorant-binding proteins that may play a role in host feeding preference.

**Conclusions:**

Our study shows that inversions are abundant and widely shared among subspecies of *Aedes aegypti* and that introgression has occurred in regions of secondary contact. This library of 32 novel chromosomal inversions demonstrates the capacity for linked-read sequencing to identify previously intractable genomic rearrangements and provides a foundation for future population genetics studies in this species.

## Background

Since the early 1920s, chromosomal inversions have been a rich source of insight into speciation and adaptation. First discovering inversions as cytogenetic markers of divergence between related *Drosophila* species, Sturtevant correctly predicted that inversions would suppress meiotic recombination within their bounds [1], causing karyotypes to diverge even in the absence of selection. The selective pressures acting on inversions have been shown to include cases of divergent selection and balancing selection (summarized in Wellenreuther and Bernatchez [2]), with often conflicting selective pressures dependent on the alleles captured in an inverted region. What is apparent is that, by allowing the co-adaptation of linked genes, inversions can contribute to local adaptation [3]. For example, associations between inversions and climatic clines have independently developed on multiple continents in various *Drosophila* species [4]. Indeed, these linked blocks of co-adapted genes can enable rapid adaptation to novel environments by selecting from a stock of globally shared standing variation—as has been seen during colonization of independent freshwater habitats by marine stickleback populations [5]. These “supergenes” can also introgress between closely related species leading to the transfer of distinct phenotypes across species boundaries [6]. Moreover, inversions are commonly implicated in the speciation process [7, 8]. Although the precise mechanisms of speciation remain a matter of debate [9–12], fixed inversions are more commonly found between sympatric sister species than allopatric [9] and both underdominance of heterokaryotypes as well as inversion-associated assortative mating may play a role in speciation [13].

Inversions are commonly studied in malaria mosquito vectors. Mosquitoes are often found as complexes of closely related species that are distinguished by fixed inversions but maintain some degree of gene flow [14], while structured populations at the subspecies level have also been posited based on differing inversion polymorphisms [15]. Chromosomal inversions have also been directly associated with phenotypes that are important for vector competence: thermal tolerance underlying range expansion [16–18], feeding behavior [19, 20], oviposition site preferences [21, 22], insecticide resistance [23], and immune response to parasites [24, 25].

However, despite their utility in other dipterans, studies of chromosomal inversions have not yet been undertaken in the mosquito *Aedes aegypti*. This is perhaps a surprise since the population structure of this organism is not just of academic interest, but could be of key importance for global health. *Ae. aegypti* is the principal vector of many viral and parasitic diseases and is found throughout the tropics [26]. Along with this global distribution comes a similarly broad dispersal of *Aedes*-borne viruses. The most common, dengue, infects millions of people per year [27] and causes more than 20,000 deaths [28], while emergent *Aedes*-borne viruses such as Zika are capable of rapid global spread [28, 29]. While chromosomes have been directly observed in Aedine mosquitoes, resolution of chromosome banding patterns is relatively poor [30, 31]; low levels of replication in polytene chromosomes and a tendency to break rather than cleanly separating mean that as little as 4% of chromosome preparations show sufficient resolution to identify banding patterns [31] and no studies have so far been able to capture visual confirmation of chromosomal inversions via banding patterns for *Ae. aegypti*.

Detecting inversions in *Ae. aegypti* via conventional sequencing-based methods is also difficult. The *Ae*. *aegypti* genome is significantly larger than other well-studied dipterans (~ 1.25 Gb), being more than five times the size of *Drosophila melanogaster* (~ 180 Mb [32]) or *Anopheles gambiae* (~ 250 Mb [33]) despite having only around 20% more genes [34]. Much of this extra genome consists of transposable elements and repeats, which comprise 65% of the *Ae. aegypti* genome. The abundance of mobile elements is a significant problem for sequencing-based detection of chromosomal rearrangements: whole-genome resequencing experiments will effectively waste more than half of the sequence they produce on intractable repetitive sequence and the mobility of these elements can lead to spurious signals of structural variation. Paired-end methods of inversion detection generally rely upon accurate mapping of reads separated by 300–500 bp and are thus poorly adapted to finding inversion breakpoints where they are buried within repetitive sequence [35, 36]. For this reason, much of the current evidence for inversions in *Ae. aegypti* is indirect: inversions are inferred from decreases in recombination rate around the putative locus. This technique has been used to detect an inversion in the sex-determining “M locus” [37] and a number of large inversions on all three chromosomes in populations in Senegal [38]. While optical and physical mapping has shown capacity to confirm large inversions [34, 39], direct confirmation of the breakpoints, or mapping the coordinates of these inversions has not proven possible. A method of detecting chromosomal inversions via sequencing would therefore be a major advance, enabling the detection of inversions that are obscure to cytogenetic methods.

Despite the challenges of working with the *Ae. aegypti* genome, there have been a number of insights into Aedine population structure. While relatively homogeneous in most of the tropics, within sub-Saharan Africa, the species exhibits notable phenotypic diversity: in contrast to the anthropophily of the global *Ae. aegypti aegypti* (*Aaa*), across sub-Saharan Africa, the generalist *Ae. aegypti formosus* (*Aaf*) dominates. Isolated regions in which both *Aaa* and *Aaf* forms are present have been found along the coast of East Africa [40, 41] and possibly in northwest Senegal [42], in which the two subspecies display varying degrees of reproductive isolation, with more hybridization in urban settings where the two forms overlap [43]. These subspecies may be expected to segregate different complements of inversions.

**Fig. 1:**
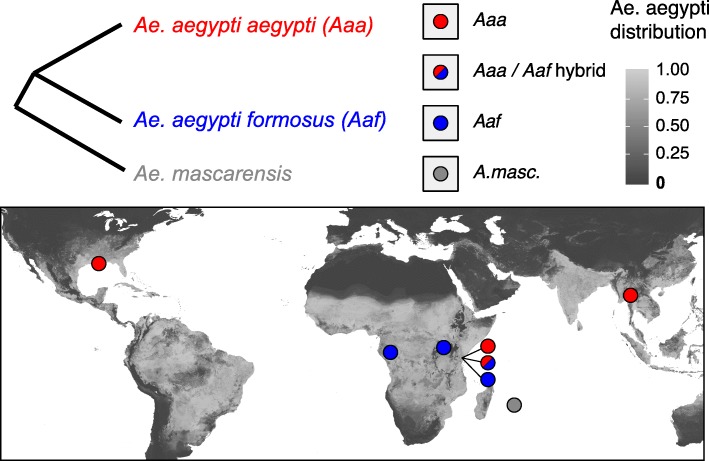
Colonies were selected to represent the extreme latitudes of both subspecies of *Aedes aegypti*: *Ae. aegypti formosus* (*Aaf*) found only in sub-Saharan Africa and *Ae. aegypti aegypti* (*Aaa*) found worldwide. Three colonies, consisting of pure *Aaa*, *Aaf*, and *Aaa/Aaf* hybrids, were founded from Rabai, Kenya: a zone of secondary contact between the two subspecies. An outgroup colony, *Ae. mascarensis*, was included from Mauritius

In order to identify inversions within the *Aedes* genome, we have employed long-read (Pacific Biosciences and Oxford Nanopore) sequencing and linked-read (10X Genomics) sequencing, in which sequencing library constructs deriving from a common DNA molecule up to ~ 80 kb in length share a common barcode, enabling one to identify reads deriving from physically proximal sequences within the genome over a greater distance than is practical with standard paired-end sequencing libraries. Regions that are not proximal in the reference assembly, but share large numbers of barcodes, are strong candidates for structural rearrangement in the genome. This technology allows us to call both inversions and SNPs with the potential to link inversion polymorphisms to more tractable SNP markers. Cataloguing inversions in this manner not only is of utility for future studies of population structure in *Ae. aegypti*, but may also highlight regions that underlie the maintenance of reproductive isolation in the two subspecies. We applied this linked-read technology to 26 individual mosquitoes from 9 different colonies (Fig. 1), generating more than 500 candidate inversions; through rigorous validation of breakpoints using both sequencing types, we were able to confirm 32 of these in multiple independently analyzed samples. The majority of these inversions are “microinversions” (those below the cytological limit of detection—here considered to be below 500 kb). The microinversions we detected are widely shared between subspecies and some occur in regions exhibiting introgression with a sister species, *Aedes mascarensis*.

## Results

More than 560 candidate inversions were called across all 28 samples (Fig S1); 465 were called by Long Ranger and 142 by GROC-SVs, with only 44 called by both. The size profile of candidates was also different between the two packages, with GROC-SVs calling larger inversions overall. There were 363 inversions called in only one sample (319/37/7 singletons called in Long Ranger/GROC-SVs/both, respectively).

**Fig. 2:**
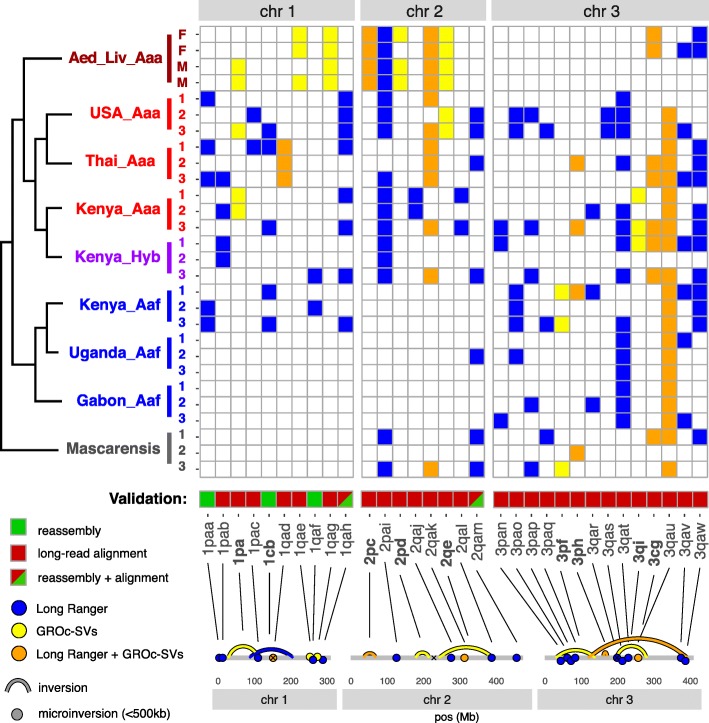
Two structural variant callers were implemented on the same linked-read data, GROC-SVs and Long Ranger. Validation of candidate inversions was performed via long-read alignment across breakpoints, or de novo reassembly of breakpoint regions. Thirty-two inversions (9 inversions, 21 microinversions) could be confirmed in both breakpoints; extensive sharing of inversions between subspecies as well as between *Ae. aegypti* and *Ae. mascarensis* is seen for inversions on chromosomes 2 and 3. Notably, chromosome 1, the homomorphic chromosome that is the site of the sex-determining locus, shows no shared inversions between subspecies outside of the hybrid zone of Rabai

Due to the highly repetitive nature of this genome, and in particular the likelihood that any active transposons will generate false-positive inversion candidates, we took an aggressive approach to validation. Inversions were considered valid if we were able to reconstruct both inverted breakpoints—either via de novo reassembly of the breakpoint regions or by alignment of long-read sequence across the breakpoints. Reconstruction of both breakpoints was limited to 32 inversions (9 large inversions and 23 microinversions) (Fig. 2): 4 confirmed via reassembly, 24 by long-read alignment, and 4 by both methods; all nine large inversions were confirmed via long-read alignment. Microinversions are here defined as any inversions under 500 kb; median size was 43 kb.

### Phylogenetic results support divergence of *A. aegypti aegypti* from *A. aegypti formosus*

Divergence as measured by *F*_st_ was high between samples from all regions, with no difference whether we were comparing within or between subspecies (mean *F*_st_ within, 0.18; between, 0.19), likely representing the effect of colonization rather than allele fixation in wild populations (Fig S2). Consistent with its emergence from an Aaf ancestral population, diversity was significantly lower in *Aaa* colonies than in *Aaf* colonies, even after removing long runs of homozygosity (*π* Thai = 3.3e^−3^, USA = 4.0e^−3^, Uganda = 4.6e^−3^, Gabon = 5.7e^−3^; mean *π Aaa* 3.9e−3, *Aaf* 5.1e^−3^; Wilcoxon rank-sum test, *P* < 2.2e^−16^) (Fig S3).

A maximum parsimony phylogeny was generated from biallelic SNP data including all colony samples and two outgroup species (*Ae. albopictus*/*Ae. bromeliae*) (Fig S4). The phylogeny showed strong support for the separation of the *Aaa/Aaf* subgroups, with all bootstrap values between *Aaa* colonies above 40%, all *Aaf* colonies at 100%, and the branching of *Aaa* from *Aaf* or *Ae*. *aegypti* from *Ae. mascarensis* also supported by 100% of bootstrap replicates. All of these results are consistent with previous studies showing that *Aaf* is the ancestral population and founder effects have led to significantly reduced diversity of *Aaa* outside sub-Saharan Africa.

### Pervasive introgression is found between subspecies and species

**Fig. 3:**
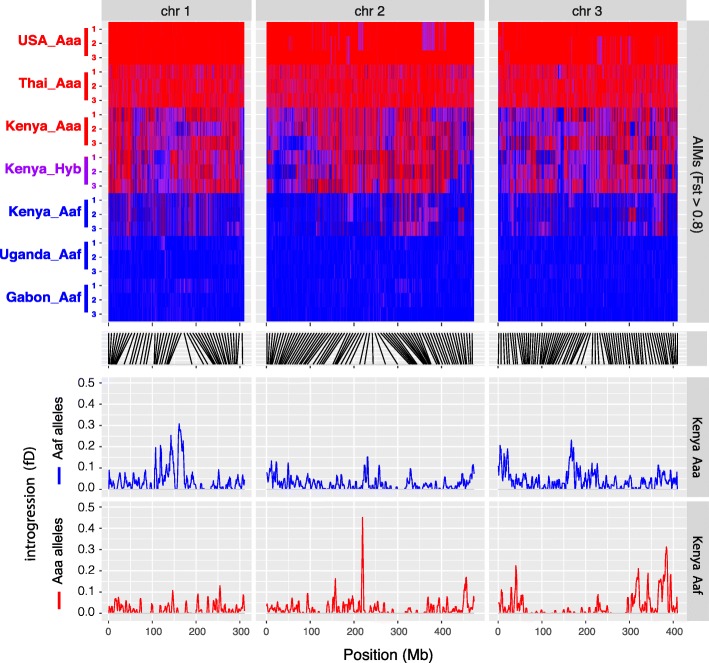
Introgression was assessed via Patterson’s *D* between global populations of *Aaa*/*Aaf* and colonies within Rabai, illustrating bidirectional gene flow between the two forms. Local peaks of introgression were identified using Martin’s *ƒ*_*D*_. Ancestry-informative markers were selected as those with *F*_*st*_ > 0.8 between all pure *Aaa*/*Aaf* colonies and illustrate the assymetric nature of introgression in this region, with a higher proportion of *Aaf* alleles found within the *Aaa* and hybrid colonies than vice versa

All three Kenyan colonies derive from mosquitoes collected in the Rabai region of Kenya. Both subspecies live in sympatry in this region and produce viable offspring after hybridization, creating an opportunity for genetic introgression between diverged populations. To test for this, we performed two tests of introgression: Patterson’s *D* for genome-wide introgression using block jackknifing to assess significance, and Martin’s *ƒ*_*D*_ to localize the genomic regions that have introgressed. At the subspecies level, tests were performed comparing Kenyan colonies to the “pure” colonies from other regions; significant introgression was detected between *Aaf* and Kenyan *Aaa*, and *Aaa* and Kenya *Aaf*—suggesting bidirectional gene flow between the two subspecies in Kenya (Fig S5). The *Kenya_Aaa* and hybrid colonies showed a similar distribution of “*Aaf*” ancestry-informative markers (AIMs); in a comparison of 10,000 AIMs, 31.57% of the AIM loci show a predominantly *Aaf* allele in the Kenya *Aaa* colony with 35.78% in the “hybrid” colony (Fig. 3), suggesting that gene flow is extensive in this region and “pure” *Aaa* is likely to be rare—consistent with a collapsing *Aaa* population at the time of sampling [C. McBride—personal communication]. Despite efforts to identify pure *Aaa* and hybrids, the two colonies appear to be sampling from the same population.

Though gene flow is clearly bidirectional in Kenya, the degree of introgression into each subspecies is not equal—whether measured by *ƒ*_*D*_ or by examining AIMs, patterns of introgression in *Aaa* and *Aaf* colonies are markedly different (Fig. 3). Compared to more than 30% of Aaf markers in the Kenya_Aaa colony, only 12.90% of these loci show a majority *Aaa* allele in Kenya *Aaf*. The patterns of introgression are also different; in Kenya *Aaa* and Kenya Hyb colonies, introgression peaks and *Aaf* alleles are present across broad swaths of the genome, including the entire chromosome 1 centromere which encompasses the sex-determining locus. In contrast, *Aaa* alleles in the *Aaf* colony are limited to short haplotypes—potentially including genes under positive selection in *Aaf*. This pattern is consistent with asymmetric introgression; however, caution should be used when making inferences from a small number of founders for the *Aaa* and Hyb colonies (Table 1) as capture of *Aaf* individuals in the *Aaa* and Hyb founders could give similar results.

**Table 1:** Colonies

Colony	Spp./sub-spp.	Collection year	Collection location	Gens in colony	No. founders	Previously published
Liverpool_Aae	*Ae. aegypti aegypti*	–	–	Unknown*	–	Matthews et al. [34]
USA_Aae	*Ae. aegypti aegypti*	2014	New Orleans, USA	7	4 females	Powell and Evans [44]
Thailand_Aae	*Ae. aegypti aegypti*	2015	Muang district, Thailand	3	> 500 larvae	
Kenya_Aae	*Ae. aegypti aegypti*	2009	Rabai, Kenya	19	3 females	McBride et al. [40]
Kenya_Hyb	*Aaa/Aaf hybrid*	2009	Rabai, Kenya	14	2 females	McBride et al. [40]
Kenya_Aaf	*Ae. aegypti formosus*	2009	Rabai, Kenya	11	9 females	McBride et al. [40]
Uganda_Aaf	*Ae. aegypti formosus*	2015/2016	Zika, Uganda	4	~ 100 inds, from 2 ovitraps	
Gabon_Aaf	*Ae. aegypti formosus*	2016	Franceville, Gabon	2	~ 10 mated females from multiple sites	
Mascarensis	*Ae. mascarensis*	2014	Mauritius	2	10 females	

*The Liverpool colony was founded circa 1935 and has undergone a large but unknown number of generations. The precise collection location and numbers of founders are not known. Our samples are the same generation used in Matthews et al. [34]

Interestingly, AIM analyses also showed a mixture of *Aae* and *Aaf* alleles in the Liverpool sequencing colony used for the genome assemblies [33, 34] with 44.63% of AIMs having a predominantly *Aaf* genotype in the Liverpool colony (Fig S6), consistent with a previously reported west African origin for *Aae* [42].

**Fig. 4:**
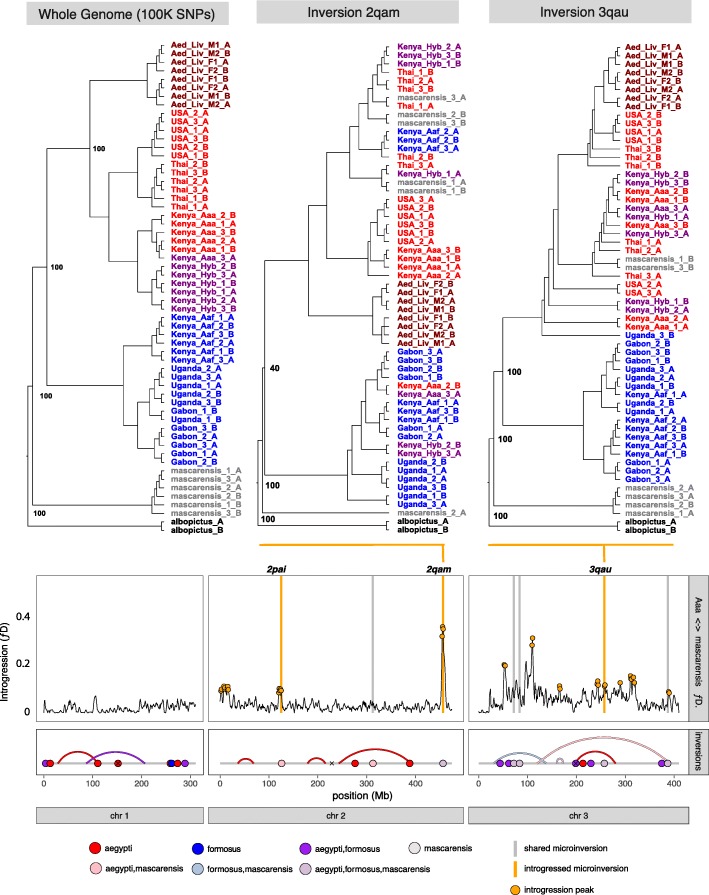
Evolutionary history of many microinversions differed from the consensus genome phylogeny; while genome-wide SNP panels showed uniform support for two separate *Aaa*/*Aaf* clades and *Ae. mascarensis* as an outgroup after 100 bootstrap replicates, phylogenies derived from the 1 MB around introgressed inversions (surrounding region showed *ƒ*_*D*_ > 1.5× IQ range and > 90% of local 200 Mb maximum) can illustrate introgression of haplotypes between diverged forms. Inversions 2qam and 3qau both show *Ae. mascarensis* haplotypes that cluster within the *Aaa* clade, indicating introgression from global Aaa populations into the local *Ae. mascarensis* populations

While introgression between sympatric populations of the same species in Kenya may not be surprising, gene flow is also detected between different species: comparing our two subspecies, and *Ae. mascarensis* with a more distant outgroup *Ae. albopictus* (Fig S5b), post-speciation gene flow was detected between our *mascarensis* colony and global *Aaa* samples. Much like the *Kenya_Aaf* samples, introgression into the *Ae. mascarensis* colony was limited to short haplotypes (Fig S5b); indeed, many of these short *Aaa* haplotypes appear to have introgressed into both Kenyan *Aaf* and *Ae. mascarensis* (Fig. 4).

### Inversions in *Aedes aegypti* do not appear to act as speciation islands

Though inversions have been proposed as speciation islands in other species [9, 11, 12], none of the 32 inversions we detected appear to be playing this role. No inversions were fixed between the *Aaa* and *Aaf* subspecies, and in most cases, *F*_st_ was not elevated within inversion regions (mean *Aaa/Aaf F*_st_ genome-wide, 0.077; inverted regions, 0.080). Microinversions with more than one gene exhibited increased *F*_st_ compared to uninverted regions, and four inversions individually showed elevated *F*_st_ between subspecies (1pab, 1pac, 1qag, 3qau) (Fig S7); however, two of these (1pac, 1qag) contain no genes; one (1pab) is present only on three haplotypes and only in Kenya; one (3qau) segregates widely across both subspecies. While we cannot rule out 1pab or 3qau being speciation islands, there is little evidence this could derive from inversion-related suppression of recombination.

### Regions containing microinversions have introgressed between populations

Comparing the locations of microinversions with the peaks of *ƒ*_*D*_, we frequently find that the two overlap. Of the 23 confirmed microinversions, 6 are found within peaks of *ƒ*_*D*_ indicating that microinversions themselves have introgressed between divergent populations.

The direction of that introgression can also be discerned by examining haplotypes within those inversions. Whereas the genome-wide phylogeny branches into clear *Aaa/Aaf* clades, phylogenies generated using only SNPs within or proximal to inversions frequently do not. One microinversion (2qam) shows haplotypes from *Aaf* individuals that are sited within an otherwise distinct *Aaa* clade; a clear indication of that a haplotype has introgressed from *Aaa* to *Aaf* in Rabai. Similarly, microinversion phylogenies indicate that a further 3 inversions (2qam, 3qas, and 3qau) have all introgressed from *Aaa* into the *Ae. mascarensis* colony in Mauritius (Fig S8). Introgression between Aaa and *Ae. mascarensis* exhibited significantly elevated *f*_*D*_ within inverted regions (mean *f*_*D*_ genome, 0.034; inversion, 0.043; Mann-Whitney *P*, 2.5e^−4^) (Table S2).

Though this study is not powered to detect associations of these inversions with specific phenotypes, the maintenance of introgressed inversions in multiple colonies could be because they confer a selective advantage. Examination of the genes within these inversions may therefore indicate potential functional consequences of particular inversion alleles.

We observed 11 microinversions containing no genes within the inverted regions, which are likely to be selectively neutral. A further six inversions contain single genes in which the increased linkage disequilibrium derived from the inversion itself is unlikely to provide any selective advantage (though one of these, 2qam, has introgressed from *Aaa* into both *Aaf* and *Ae. mascarensis* and would be an intriguing target for functional characterization). The remaining 15 inversions contained more than two genes and could facilitate co-adaptation of genes. Intriguingly, inversion 3qau contains ten genes, eight of which comprise a family of odorant-binding proteins, of which two (OBP11/OBP65) have previously been shown to be differentially expressed in zoophilic/anthropophilic colonies [40]. A full list of genes in inversions and proximal regions is in Table S2 and divergent non-synonymous markers in 3qau in Table S3.

## Discussion

Using a combination of linked-read and long-read sequencing platforms, we have generated whole-genome sequences of 26 individual *Aedes* mosquitoes representing the extreme longitudes of the global distribution of the *Aaa* and *Aaf* subspecies. These technologies allowed us to detect both single-nucleotide and inversion polymorphisms from the same library and have allowed us to generate a catalogue of inversions based on fully independent detection of structural variants in each individual. This is the first large-scale survey for inversions in this species and a crucial first step in establishing the distribution of these variants in wild populations. It is important to note that the 23 novel microinversions that we have identified would likely not have been detectable by either traditional cytology or short-read sequencing approaches.

The phylogeny we generated based on SNP genotypes supports previous conclusions regarding the origins of the *Aaa* and *Aaf* subspecies and supports their genetic divergence [42, 43]; however, we did not detect any inversion that was fixed between *Aaa* and *Aaf* colonies. Most inversions segregated widely among colonies despite the large geographic distances separating the founders and elevated *F*_*st*_ was not found within most inversions. Tests for introgression showed that microinversions are commonly found within introgressed regions and that the distribution of microinversions in Kenya derives, at least in part, from introgression between subspecies.

Few of the more than 500 inversion candidates were validated by breakpoint reassembly or long-read sequence (Table 2). Indeed, few were reliably found in both technical replicates of the “Liverpool” samples, indicating that even with the advantage of long-range linking information, reliably calling inversions is challenging in *Aedes* spp. We cannot rule out the possibility that inversions are more plentiful but repetitive sequence flanking the breakpoints prevents us from identifying or validating them, or that breakpoints remain improperly assembled in the reference assembly as they are in anopheline genomes [45].

**Table 2:** Inversion calls

Inversion	Position	Size	Micro	Prog	Validation
1paa	1:3662097-3696373	34276	*	Long Ranger	Assembly
1pab	1:12833450-12864379	30929	*	Long Ranger	Long-read align
1pa	1:28526219-110482104	81955885		GROC-SVs	Long-read align
1cb	1:86573078-207047458	120474380		Long Ranger	Long-read align
1pac	1:110426504-110463452	36948	*	Long Ranger	Assembly
1qad	1:151766620-151847533	80913	*	Both	Long-read align
1qae	1:258805045-258838552	33507	*	GROC-SVs	Long-read align
1qaf	1:261838607-261877216	38609	*	Long Ranger	Assembly
1qag	1:273812946-273859121	46175	*	GROC-SVs	Long-read align
1qah	1:288864540-288902671	38131	*	Long Ranger	Both
2pc	2:36263327-68515898	32252571		Both	Long-read align
2pai	2:125768796-125807158	38362	*	Long Ranger	Long-read align
2pd	2:178843909-215734436	36890527		GROC-SVs	Long-read align
2qe	2:242868946-392168275	149299329		GROC-SVs	Long-read align
2qaj	2:276157193-276249519	92326	*	Long Ranger	Long-read align
2qak	2:312677653-312836936	159283	*	Both	Long-read align
2qal	2:387870950-387930807	59857	*	Long Ranger	Long-read align
2qam	2:456306790-456341898	35108	*	Long Ranger	Both
3pf	3:30056138-138778486	108722348		GROC-SVs	Long-read align
3pan	3:43852147-43896847	44700	*	Long Ranger	Long-read align
3pao	3:61571542-61611075	39533	*	Long Ranger	Long-read align
3pap	3:72073163-72216512	143349	*	Long Ranger	Long-read align
3paq	3:83753676-83794150	40474	*	Long Ranger	Long-read align
3cg	3:119518403-392983952	273465549		Both	Long-read align
3ph	3:161230873-171356784	10125911		Both	Long-read align
3qi	3:196536178-280271694	83735516		GROC-SVs	Long-read align
3qar	3:198751700-198836215	84515	*	Long Ranger	Long-read align
3qas	3:213245322-213670971	425649	*	Long Ranger	Long-read align
3qat	3:229688967-229782178	93211	*	Long Ranger	Both
3qau	3:257058020-257252866	194846	*	Both	Both
3qav	3:374671141-374714093	42952	*	Long Ranger	Assembly
3qaw	3:386773996-386844766	70770	*	Long Ranger	Long-read align

Nevertheless, the size and distribution of the inversions we called are unexpected. Prior studies had indicated large inversions around the centromere of chromosomes 1, as inferred due to the suppression of recombination seen between *Aaa* and *Aaf* [37, 38]; Dickson et al. also identified rearrangements of BAC clone markers indicating a pair of pericentromeric inversions on chromosome 3 and a large inversion on the 2p arm proximal to the chromosome 2 centromere and suggested that these rearrangements could be linked to a reduction in fecundity in Senegalese *Aaf* [39]. While we found inversions that may be consistent with the positions of these rearrangements (1cb, 2pd, 3cg), we did not detect any that were fixed or at high frequency in *Aaa*, none of these inversions showed elevated *Aaa*/*Aaf* divergence, and none was found in *Aaf* (fixation of these inversions in the reference *Aaa* strain would be detected as a polymorphism in the *Aaf* subspecies).

If the absence of large fixed inversions between *Aaa* and *Aaf* were confirmed, we would require an alternative explanation for the reduced recombination around the chromosome one centromere in *Aaa/Aaf* crosses identified by previous studies [38]. The extensive repeat structure could provide one possible mechanism for this reduced recombination. *Aedes aegypti* has long been characterized as a highly repetitive, short-period interspersion species [46], and differing complements of transposable elements or satellite lengths could act to reduce collinearity. The extent to which microinversions themselves suppress recombination is also not known; while it has been shown previously that large inversions act to suppress recombination up to 1.5 Mb outside of the inversion breakpoints [47], this work has not been applied to microinversions and the extent to which the lack of collinearity suppresses recombination is unknown.

The nature of introgression in Kenya is also instructive. Clear asymmetry is seen in the distribution of AIMS in these colonies with far more *Aaf* alleles are found in the *Aaa* colony than vice versa. Similar patterns have been seen in structured populations of both anopheline [48] and culicine [49] mosquitoes where this asymmetric pattern of backcrossing is thought to underlie divergence in the face of extensive hybridization [50]. Both the geographical and genomic distributions of inversions support this conclusion of hybridization between cosmopolitan *Aaa* and local populations in Kenya or closely related species in Mauritius. Phylogenetic analysis of inversion-linked SNPs further suggests that, although the predominant direction of gene flow in the Kenyan hybrid zone is from *Aaf* to *Aaa*, the predominant direction of gene flow within inversions is from synanthropic *Aaa* populations to *Ae. mascarensis* and sylvan *Aaf* populations. This may be indicative of adaptive introgression; however, caution should be taken when inferring that this is due to inversions—particularly as the strongest signal of introgression (2qam) contains only one gene and would derive little or no selective advantage from being sited within an inversion.

Indeed, caution must be taken when interpreting these population genetic signals from colony samples. The reduced haplotypic diversity in colonies relative to wild populations provided a valuable opportunity to validate the novel inversion calling approaches we employed through replicated calls. However, unlike samples taken directly from the field, these samples will be subject to allele loss deriving from the colonization process itself [51]. Signals such as the apparent asymmetric introgression could derive from stochastic loss of haplotypes or from neutral selection acting upon different population sizes [52] (*Aaa* was rare in this area when colony founders were collected (McBride pers. comm*.*)). Small founder numbers (Table 1) also require caution when inferring introgression signals within these colonies or the sampled founders are representative of the wider population. While signals such as the introgression of inversion 2qam into both *Aaf* and *Ae. mascarensis* populations (colonies that were collected more than 2000 km apart and maintained in different laboratories) are difficult to explain other than via true introgression, confirmation of all of these signals will ultimately require prospective population genetic projects in *Aedes aegypti*.

Nevertheless, adaptive introgression has been seen in other vector mosquitoes, where it was responsible for the transfer of advantageous traits between sister species. For example, in sympatric populations of *Anopheles gambiae* and *Anopheles coluzzii*, insecticide resistance alleles appear to have introgressed between *An. gambiae* and *An. coluzzii* [53, 54], and historical introgression of large inversions may also have occurred between more distantly related taxa [14, 55], with the potential to transfer adaptive traits underlying range expansion into xeric environments [17, 56]. Examining genes within introgressed inversions can therefore generate testable theories as to which phenotypes might be under positive selection. In many cases, our microinversions have not captured any genes within the inverted region itself, and are likely to be selectively neutral, or contain only single genes which would gain little advantage from suppression of recombination; however, some have captured more than one gene (Table S2).

The microinversion that has captured the most genes is 3qau, which encompasses 10 genes, eight of which are confirmed or putative odorant-binding proteins (OBPs). OBPs are short (typically < 20 KDA) proteins that are thought to bind and solubilize small hydrophobic molecules and to play some role in olfaction [57, 58]. The mechanism by which they do this is unclear; an OBP is not necessary to activate the odorant receptor complex [59]; instead, their binding of soluble odorants is thought to assist in transportation of odorants to the odorant receptor complex or to buffer olfactory stimuli enabling the olfactory response to function under varying levels of stimuli [60]. Though the specificity of OBPs to each class of odorants is yet to be determined, in two different mosquitoes, OBP1 (Ag/CqOBP1) has been shown to bind to compounds that are associated with oviposition sites [61, 62]. Whether through changes in conformation or expression, there is clear potential for these molecules to affect mosquito behavior and vectorial capacity.

Of the eight OBPs in inversion 3qau, two (OBP11/OBP65) have been demonstrated to have significantly different expression between zoophilic and anthropophilic colonies [40]. Of these two, one has an orthologue in *An. gambiae* (AaOBP11/AgOBP25) that has been shown to be expressed in mosquito antennae [63]. This inversion has introgressed from wild *Aaa* populations into the sampled *Ae. mascarensis* population after secondary contact. That this inversion is linked to increased anthropophily is a tantalizing possibility and one that bears further investigation.

Introgression of such an inversion between sympatric populations would be of more than academic interest. As has been seen within anopheline vectors, inversions can be associated with a wide variety of phenotypes important for vector competence. Even if partial reproductive isolation is maintained by asymmetric introgression, the transfer of inversions between divergent populations in secondary contact zones is likely to generate increased phenotypic plasticity within these regions and reduce our ability to reliably predict phenotypic profiles of vector species. Larval source management requires an understanding of where those larvae are oviposited: a key difference between Aaa and Aaf populations [64] and one that is linked to anthropophily [40]. Transference of anthropophilic biting tendencies to sylvan populations could impact larval control programs and lead to an increase in outbreaks of arboviral diseases. We have not detected any directional introgression from African *Aaf* into global *Aaa* populations, suggesting this may be a concern limited to regions of secondary contact between *Aaa* and *Aaf* populations. Yet regions of mixed *Aaa/Aaf* genotypes and increased phenotypic diversity include areas outside sub-Saharan Africa [43, 65] and importation of global *Aaa* is unlikely to be limited to east Africa and Mauritius, suggesting that this localized concern could become a global one.

## Conclusions

We have genotyped chromosomal inversions in eight colonies of *Ae. aegypti*, representing the extreme latitudes of each subspecies, one location where they live in sympatry, and the outgroup species *Aedes mascarensis.* Applying a combination of linked-read and long-read methods, we have detected and validated 32 novel inversions. In contrast to anopheline mosquito species in which large, ancient (predating speciation) inversions predominate, we find large numbers of microinversions. Most inversion polymorphisms are shared between subspecies and post-divergence hybridization and genetic introgression has occurred between subspecies and with *Ae. mascarensis*. If repeated in other regions of the world, this introgression of inversions could affect phenotypic diversity in *Ae. aegypti* and has the potential to impact dengue control programs.

## Methods

### Colony sampling

To add to the previously generated linked-read data from the Liverpool colony [34], we selected 8 colonies of *Aedes aegypti* representing the extreme east and west of both *Aaa* and *Aaf* as well as a hybrid zone (Fig. 1). “Pure” *Aaa* colonies were established from North America (New Orleans, USA) and Asia (Chiang Mai, Thailand) and “pure” *Aaf* colonies from West Africa (Franceville, Gabon) and East Africa (Uganda). Mosquitoes were also sampled from an *Aaa*/*Aaf* hybrid zone in Rabai, Kenya, where the two subspecies are believed to live in sympatry but represent genetically distinct units [43]; three colonies were established from these samples—one *Aaa*, one *Aaf*, and one founded from hybrids. An outgroup colony was also founded from a closely related species *Aedes mascarensis* (Mauritius). Colony sampling dates, locations, number of generations, and founders are given in Table 1*.*

### Library preparation/sequencing

High molecular weight DNA was extracted using the Qiagen MagAttract kit according to the manufacturer’s instructions with minor modifications (rapid vortexing was replaced by inversion and wide-bore pipette tips were used—both to prevent excessive shearing of DNA). DNA extracted from each individual pupa was loaded into a separate lane of the 10X Chromium for barcode tagging of the amplicons, then an Illumina library was prepared. Each resulting library was sequenced with a full lane of Illumina X10 sequencing (~ 120-Gb total output) for a target of 100-fold coverage.

### Alignment/genotyping

Sequences were aligned to the reference using BWA via the LongRanger-Align function (longranger v. 2.1.3). Variants were called using GATK HaplotypeCaller (GATK version 3.5.0) and filtered for quality (QD > 5), strand bias (FS < 60), and read position (RankSum < 8). Only biallelic SNPs were used for subsequent analyses. Previously generated 10X library sequence was included from the reference Liverpool colony as described in Matthews et al. [34].

Additional sequence sets were obtained from Genbank for two further outgroup species to allow the determination of derived alleles in our colony samples. *Aedes albopictus* (SRA project: SRP064281) and *Aedes bromeliae* (SRA project: SRP092518) were both aligned and called in the same manner. Two resultant callsets were produced: the first with SNPs that could be reliably called across all 8 of our colonies (colony callset), and the second for SNPs that could be called in all colony samples and the two outgroup species (conserved callset).

### Chromosomal inversion detection

The full Long Ranger “WGS” pipeline (Long Ranger v.2.1.5) was run on all samples, with memory overrides for both the SNP/INDEL phasing and SV calling stages required due to the high heterozygosity found in these samples. The pipeline was run with the pre-called VCF from the prior variant calling ensuring that the same sites were genotyped and phased in all samples. A second SV calling pipeline, GROC-SVs, was run on the BWA alignments generated for variant calling. Long Ranger was run with both repeatmasked and unmasked references to account for potential TE-associated false positives. Structural variants were compared between each individual and both methods and were merged if they showed a 95% pairwise overlap in position.

### Phasing

10X-phased genotypes were also generated via the Long Ranger pipeline. Haplotypes generated by Long Ranger vary in size depending on the level of heterozygosity in the region, since variants that are significantly more distant than our typical molecule size cannot be efficiently phased. In regions where one sample showed a long pair of haplotypes, haplotypes in other samples within the same colony were compared in order of descending size; a haplotype was only considered novel if it showed more than 1 SNP difference per kilobase. Regions that showed less than this degree of divergence from a longer haplotype were also assumed to derive from that same founder haplotype.

After examination of the levels of hybridization within the Rabai colonies, this was also repeated across all nine samples from Rabai, enabling reconstruction of hybrid haplotypes and more detailed examination of the introgressed genotypes.

For the conserved callset, including *A. albopictus*/*A. bromeliae*, statistical phasing was performed via SHAPEIT (v2.837) allowing us to determine phylogenies for individual haplotypes.

### Validation

Due to the large number of chromosomal inversion candidate regions detected, and the high probability of TE-related false positives, we took an aggressive approach to inversion validation in which independent confirmation of both breakpoints was required for validation. Breakpoint reconstruction took two forms: breakpoints could be reassembled de novo or long-read sequence could be aligned across the inverted breakpoint.

Long-read sequence for the Liverpool colony consisted of PacBio sequence generated during genome assembly [34], while all other colonies were sequenced using an Oxford Nanopore GridIon sequencer. Read N50 differed between the two sequencing runs (PacBio: N50 = 14,307, Nanopore N50 = 6789), most likely as a result of degradation in DNA stocks between PacBio and Nanopore sequencing runs. The longest reads (> 5 kb) were selected within each sequencing type, and competitive alignment was performed using Minimap2 (V2.11 using map-pb/map-ont presets) to a pan-genome sequence containing both the original reference breakpoints and artificially inverted breakpoints (along with the rest of the chromosome with the actual breakpoints masked). Reads were considered to align to the breakpoint if they extended at least 1 KB across the breakpoint on both sides. A second pan-genome was created consisting of 1000 artificial breakpoints that were generated to have a similar complement of repeats and transposable elements to our candidate regions; alignments to these artificial breakpoints were used to calculate the typical level of misalignment in an uninverted region with a false discovery rate of under 1% per breakpoint (1e^−4^ for both breakpoints): breakpoints with under 10X coverage or more than 2× the inter-quartile range were discarded and those that remained were considered valid if the alignment had more than 37% of long-read alignment to the inverted breakpoint.

Breakpoint assembly was performed using the linked-read-aware Supernova software (v1.1.4). In each case, all reads within 50 kb of the two candidate breakpoints were collected along with all reads linked to that region by at least one 10X barcode. This targeted sequence set was then aligned using Supernova, and “megabubble” sequences (i.e., phased supercontigs) greater than 10 kb in length were realigned to the reference using Minimap2 (v2.11). Those supercontigs aligning to either both upstream or both downstream regions of each breakpoint, with an alignment score above 60, were used to determine true inversions. False discovery rate was determined by running the reassembly script with our artificial inverted regions.

### Heterozygosity/*F*_st_/introgression/AIMs

Heterozygosity was calculated via VCFtools (v0.1.14) from the colony callset in 1-Mb windows. Long regions of homozygosity are assumed to be the result of inbreeding within colonies and were identified using the LROH function of VCFtools. Significance of heterozogosity differences between *Aaa* and *Aaf* samples was determined by Wilcoxon rank-sum test. *F*_*st*_ (divergence) was calculated between all pairs of colonies via the *F*_*st*_ function within VCFtools. Ancestry-informative markers (AIMs) for *Aaa*/*Aaf* were determined based on *F*_*st*_ between the unintrogressed colonies (i.e., Uganda + Gabon vs Thailand + USA); AIMs were chosen if they exhibited *F*_*st*_ > 0.8 and were callable in both colony and conserved datasets. Mann-Whitney tests were used to compare *F*_*st*_ between subspecies within our called inversions and within our artificial inverted regions, as well as between different classes of inversions (intergenic, monogenic, polygenic).

Genome-wide introgression was tested using Patterson’s *D* [66], and significance was determined by *Z*-score (2 or more considered significant) after calculation of standard deviation by block jackknifing. Where samples were found to have significant genome-wide “*D*,*”* localized introgressed regions were identified using Martin’s *ƒ*_*D*_ statistic, which controls for regions of low diversity [67]; peaks of introgression were defined as those with *ƒ*_*D*_ above 1.5× the IQ range and more than 90% of the local 200 Mb maxima. Mann-Whitney tests were used to compare *ƒ*_*D*_ within and outside microinversions.

## Supplementary information

**Additional file 1: Figure S1.** All Unconfirmed Structural Variants. a) Over 500 inversion candidates were detected by linked-read analysis, of which 32 (9 inversions, 21 microinversions) could be confirmed by breakpoint reassembly or long-read alignment. b) a further 210 insertion and 404 deletion candidates were discovered using linked read analysis, though without a clear method for validation of candidates, these classes of structural variant were not further investigated.

**Additional file 2: Figure S2.***F*_*st*_ Between Colonies. *F*_*st*_ values were calculated using vcftools based on the colony SNP set. Elevated *F*_*st*_ values were seen between all groups of samples following colonization, with the highest values between comparisons of USA or *Ae. mascarensis* colonies. *F*_*st*_ was uniformly lower on chromosome 3 (fig S3b) where the highest degrees of both introgression and inversion sharing were seen.

**Additional file 3: Figure S3.** Genome-wide Heterozygosity and Long Runs of Homozygosity. Genome-wide heterozygosity values are dominated large blocks of homoygosity due to inbreeding. After removal of these regions using VCFtools LRoH function, heterozygosity was seen to correlate weakly but significantly with time in colony (Pearson’s product-moment correlation r = − 0.19, *P* < 2.2e^− 16^) and was found to be lower in all pure *Aaa* colonies than *Aaf* (Wilcoxon rank-sum test, P < 2.2e^− 16^) consistent with the recent global emergence of this clade. While the Liverpool strain is shown on the figure, due to extreme inbreeding and an uncertain number of generations since its foundation in the mid 1930s, this colony was not included in any analyses.

**Additional file 4: Figure S4.** Bootstrapped Whole-Genome Phylogeny. A maximum parsimony phylogeny was derived from 10,000 genome-wide markers giving the background phylogeny to which we compared the inverted regions. Strong bootstrap support was shown for the separation between *Aaa / Aaf*, *Ae. aegypti / Ae. mascarensis*, and for the isolation of the *Ae aegypti* Liverpool colony.

**Additional file 5: Figure S5.** All Applied Introgression Tests. Patterson’s D was used to assess introgression between a potential introgressing clade and pair of putative sister clades, with an outgroup used to determine derived alleles for the other three clades; *5a)* introgression into individual colonies was compared by applying Patterson’s D statistic to each colony as compared to the other two colonies of the same subspecies (e.g we examined *Aaf* introgression into *Kenya_Aaa* vs *Thai_Aaa + USA_Aaa* using *Ae. mascarensis* as the outgroup). Specific tests applied are given in figures i,iii,v, and the results for each shown in figs ii,iv,vi. Significance was tested by block jackknifing. 5b) The same test was applied to introgression between mascarensis and any individual population (i-iv), as well as between *Ae mascarensis* and all Aaf or Aaa populations (v-vi); all inter-specific tests used *Ae albopictus* as the outgroup. 5c) Significant introgression was detected in between *Aaf* and *Kenya_Aaa*, between *Aaa* and *Kenya_Aaf*, indicating bidirectional introgression between the two subspecies in Kenya. Martin’s *f*_*D*_ statistic was applied to identify specific introgressed loci in these two populations and was also applied to testing for Aaf introgression into the ‘hybrid’ population in this region. 5d) significant introgression was also detected between *Ae. mascarensis* and global *Aaa* populations. Notably one *f*_*D*_ peaks at the distal end of chromosome 2q appeared to have introgressed between *Aaa* and *Kenya_Aaf* and *Aaa* and *Ae mascarensis.*

**Additional file 6: Figure S6.** Aegypti / Formosus Ancestrally-Informative Markers, Liverpool colony. Contrary to expectation, the *Ae. aegypti* Liverpool strain used for sequencing was not a clear *Aaa* strain, but instead demonstrated evidence of both *Aaa* and *Aaf* alleles. This is consistent with a west African origin and prior evidence from Crawford et al. of forms ancestral to Aae/Aaf in this region [40].

**Additional file 7: Figure S7.** Elevation of *F*_*st*_ Within Inversions. *F*_*st*_ between pure Aaa and Aaf colonies was calculated in 1mb windows across the genome and windows that contained either an entire microinverisons or a larger inversion breakpoint were compared to a set of artificial inversions constructed of ‘breakpoints’ containing similar levels of TEs and repeats. *F*_*st*_ was compared between categories via Wilcoxon rank-sum test. A) Of the 32 inversions 4 (shown in red) showed significantly elevated levels of *F*_*st*_ indicating a higher degree of differentiation between subspecies; three on chromosome one, the fourth inversion showing elevated *F*_*st*_ is 3Qau, containing 10 genes, 8 of which are odorant binding proteins previously seen to be differentiated between the two subspecies. B) categories of inversions: macroinversions, microinversions without genes, with one gene, and with many genes, were compared to artificial inversions; while polygenic inversions did show elevated *F*_*st*_ compared to artificial inversions, there was no significant difference found when comparing 0 and 1-gene microinversions to polygenic microinversions.

**Additional file 8: Figure S8.** Within-Inversion Phylogenies. Maximum parsimony phylogenies were derived from the 1 M region surrounding each microinversion in order to establish the unique evolutionary history of these regions. In many cases haplotypes did not cluster into clean sub-species clades, but instead indicated extensive introgression of haplotypes from global Aaa populations into local sylvatic forms.

**Additional file 9: Table S1.** fD elevation in inverted regions.

**Additional file 10: Table S2.** Microinversion genes.

**Additional file 11: Table S3.** Non-synonymous variants: Inversion 3qau.

## Data Availability

All linked-read and long-read libraries generated for this study were deposited with SRA under project ID: PRJNA559933 [68]. Linked-read and PacBio libraries used for calling and validation of the “Liverpool” samples were previously deposited under project ID PRJNA318737 [69].
