# Supplementary figures and images for: Linked-read sequencing identifies abundant microinversions and introgression in the arboviral vector Aedes aegypti
Source: BMC Biol. 2020 Mar 12;18:26. doi: 10.1186/s12915-020-0757-y (PMC7068900; doi:10.1186/s12915-020-0757-y)

**Fig S1a:**

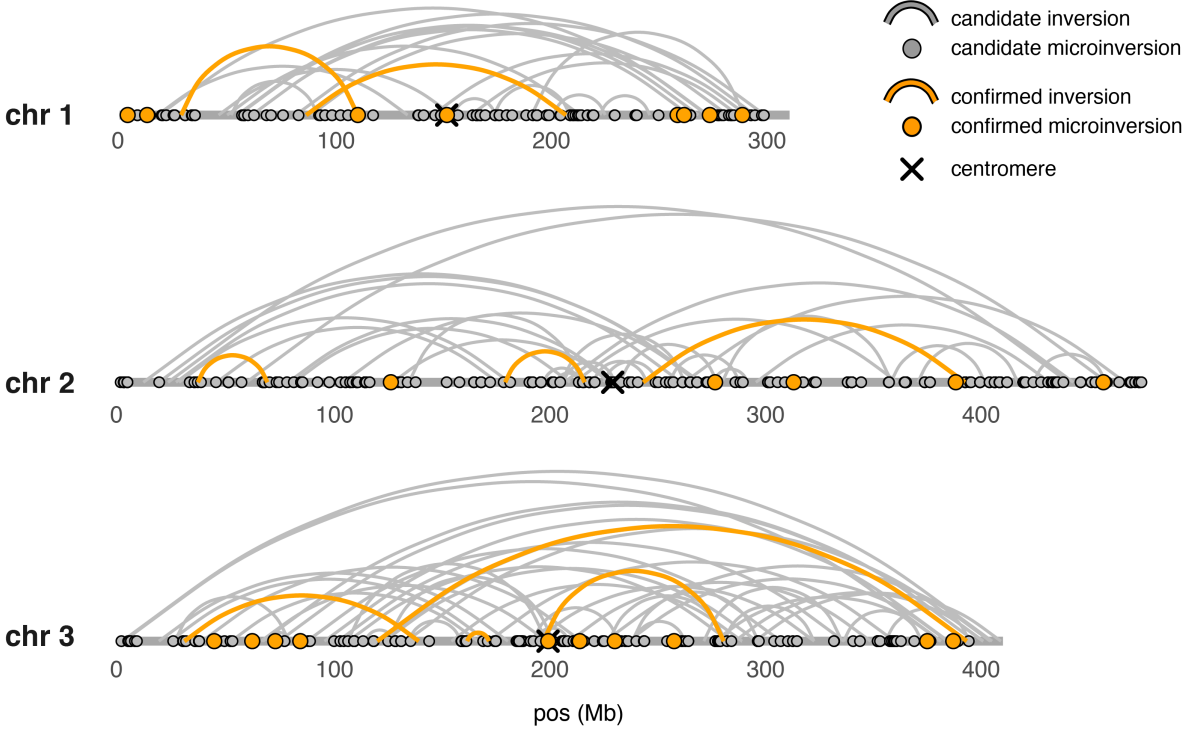

Fig S1b:

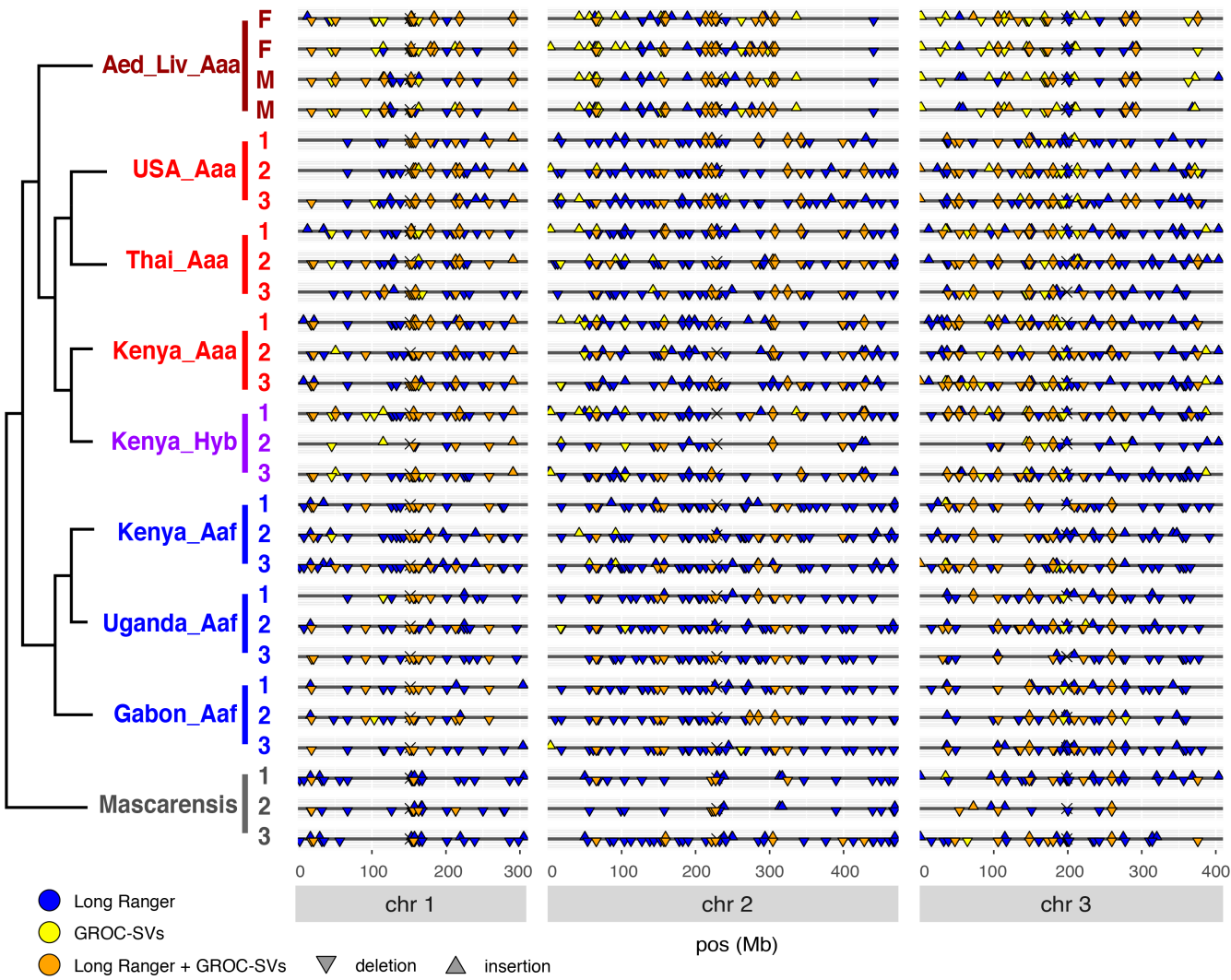

Supplement: Supplementary file 1 — Additional file 1: Figure S1. All Unconfirmed Structural Variants. a) Over 500 inversion candidates were detected by linked-read analysis, of which 32 (9 inversions, 21 microinversions) could be confirmed by breakpoint reassembly or long-read alignment. b) a further 210 insertion and 404 deletion candidates were discovered using linked read analysis, though without a clear method for validation of candidates, these classes of structural variant were not further investigated. [file 12915_2020_757_MOESM1_ESM.pdf]

Fig S2a:

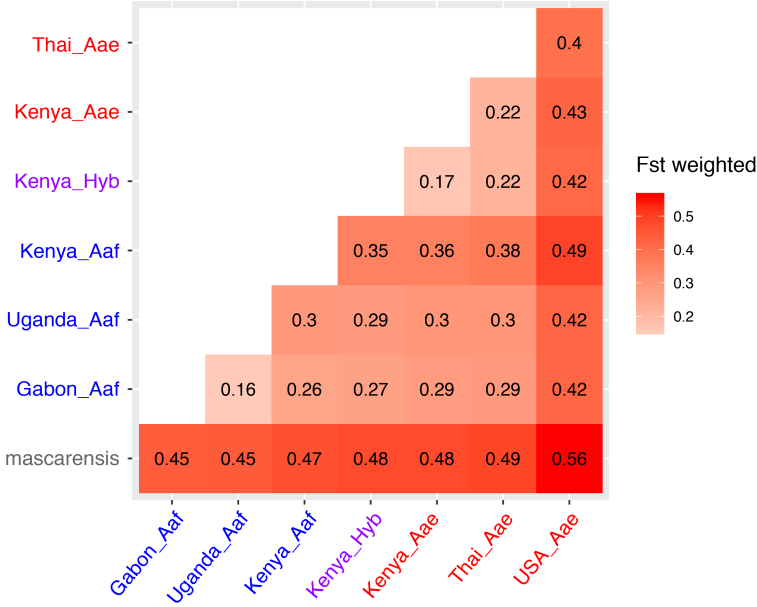

Fig S2b:

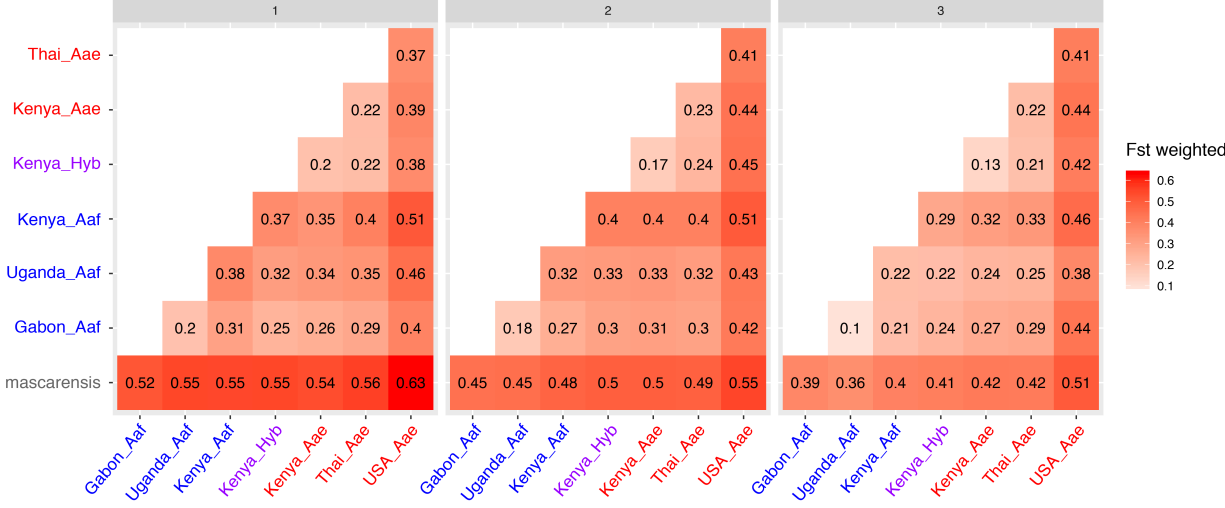

Supplement: Supplementary file 2 — Additional file 2: Figure S2.Fst Between Colonies. Fst values were calculated using vcftools based on the colony SNP set. Elevated Fst values were seen between all groups of samples following colonization, with the highest values between comparisons of USA or Ae. mascarensis colonies. Fst was uniformly lower on chromosome 3 (fig S3b) where the highest degrees of both introgression and inversion sharing were seen. [file 12915_2020_757_MOESM2_ESM.pdf]

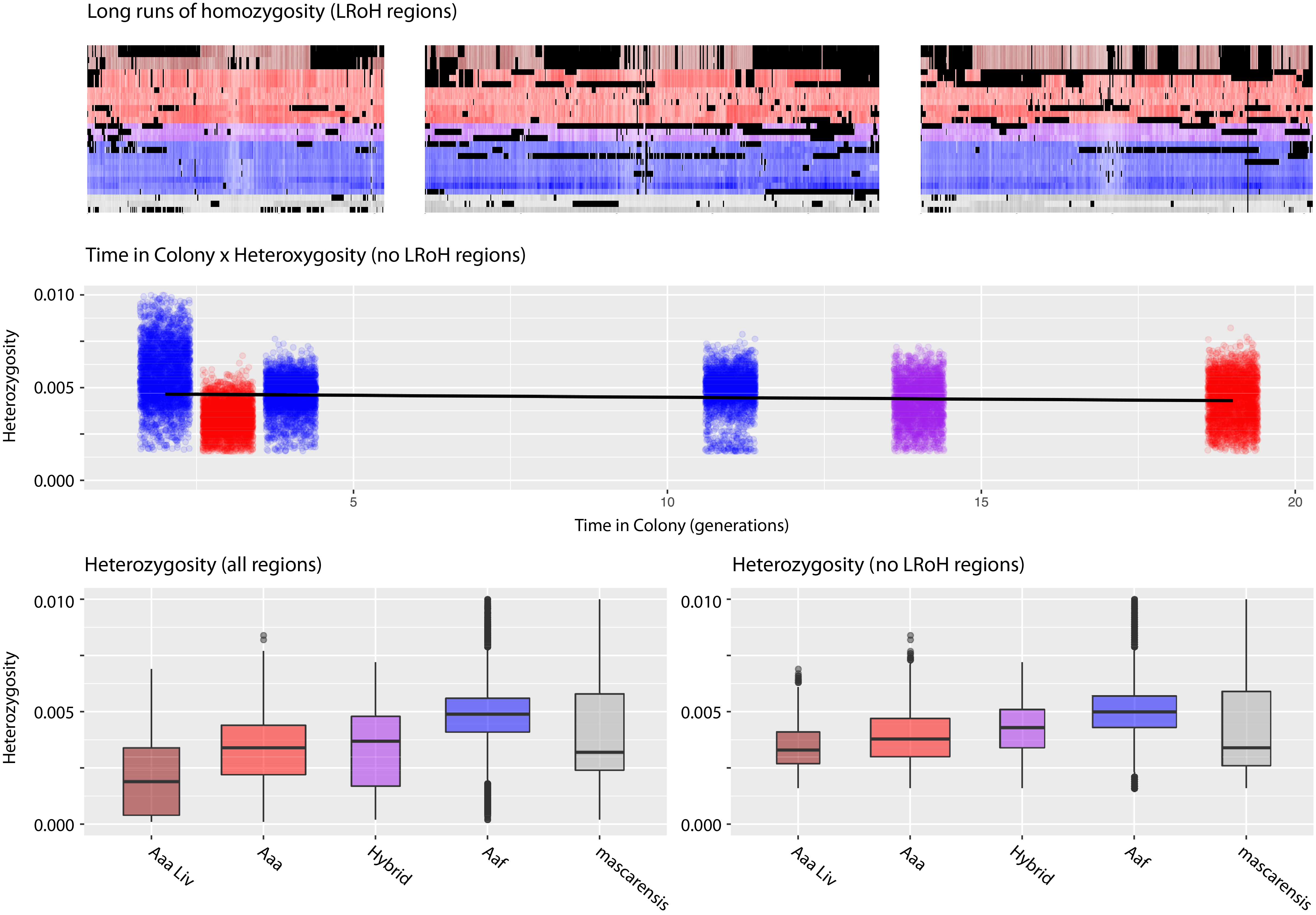

Supplement: Supplementary file 3 — Additional file 3: Figure S3. Genome-wide Heterozygosity and Long Runs of Homozygosity. Genome-wide heterozygosity values are dominated large blocks of homoygosity due to inbreeding. After removal of these regions using VCFtools LRoH function, heterozygosity was seen to correlate weakly but significantly with time in colony (Pearson’s product-moment correlation r = − 0.19, P < 2.2e− 16) and was found to be lower in all pure Aaa colonies than Aaf (Wilcoxon rank-sum test, P < 2.2e− 16) consistent with the recent global emergence of this clade. While the Liverpool strain is shown on the figure, due to extreme inbreeding and an uncertain number of generations since its foundation in the mid 1930s, this colony was not included in any analyses. [file 12915_2020_757_MOESM3_ESM.png]

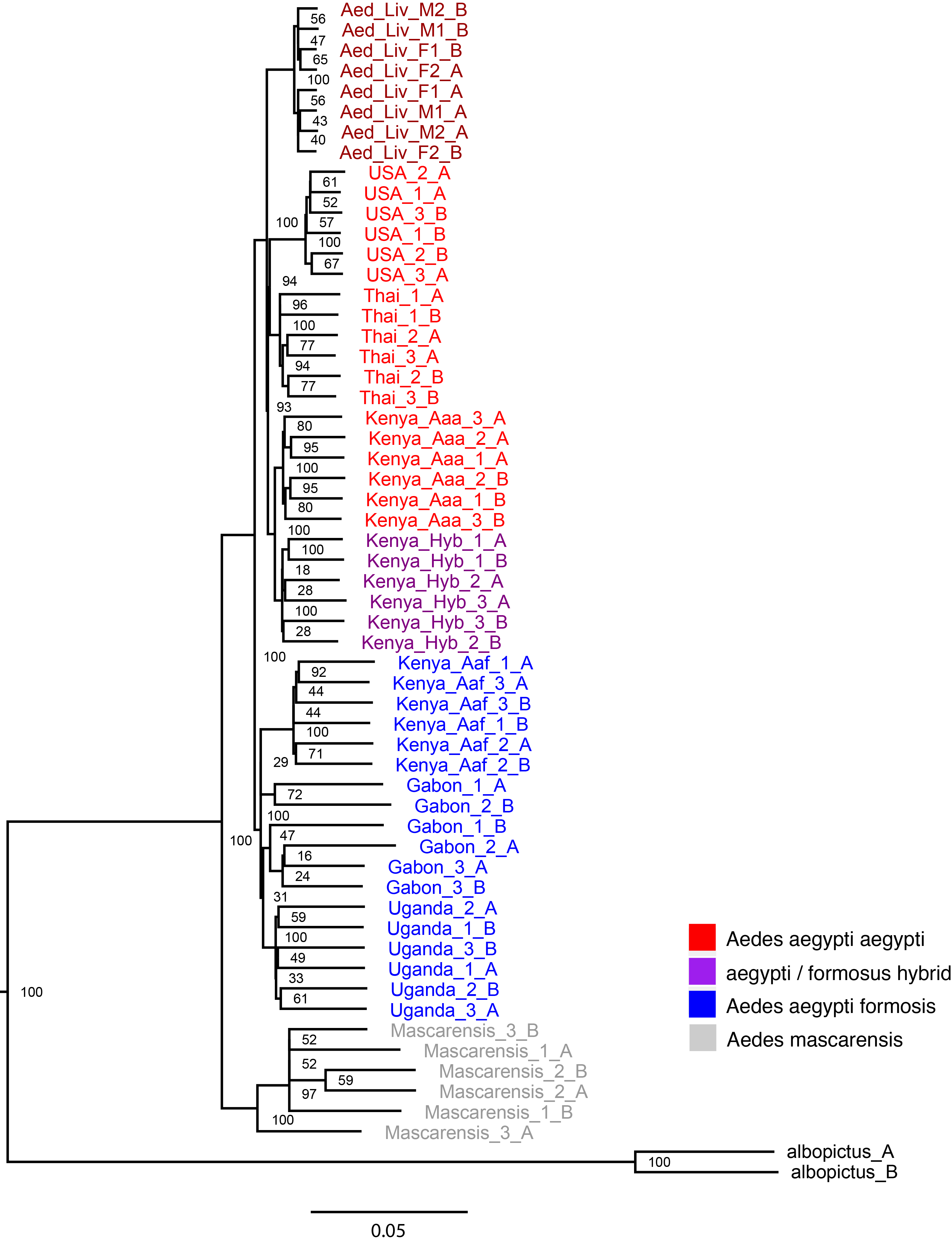

Supplement: Supplementary file 4 — Additional file 4: Figure S4. Bootstrapped Whole-Genome Phylogeny. A maximum parsimony phylogeny was derived from 10,000 genome-wide markers giving the background phylogeny to which we compared the inverted regions. Strong bootstrap support was shown for the separation between Aaa / Aaf, Ae. aegypti / Ae. mascarensis, and for the isolation of the Ae aegypti Liverpool colony. [file 12915_2020_757_MOESM4_ESM.png]

Fig S5a:

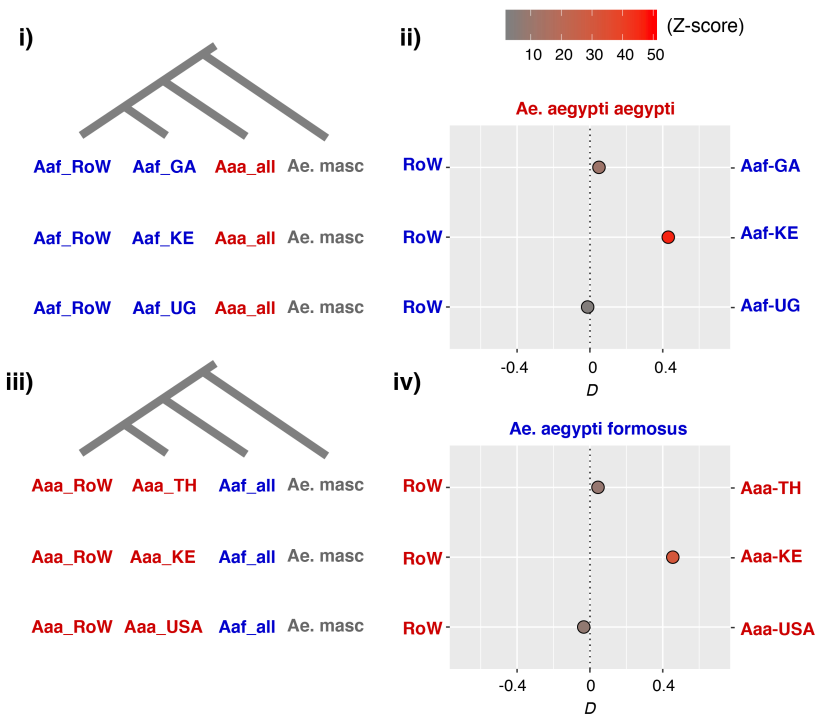

Fig S5b:

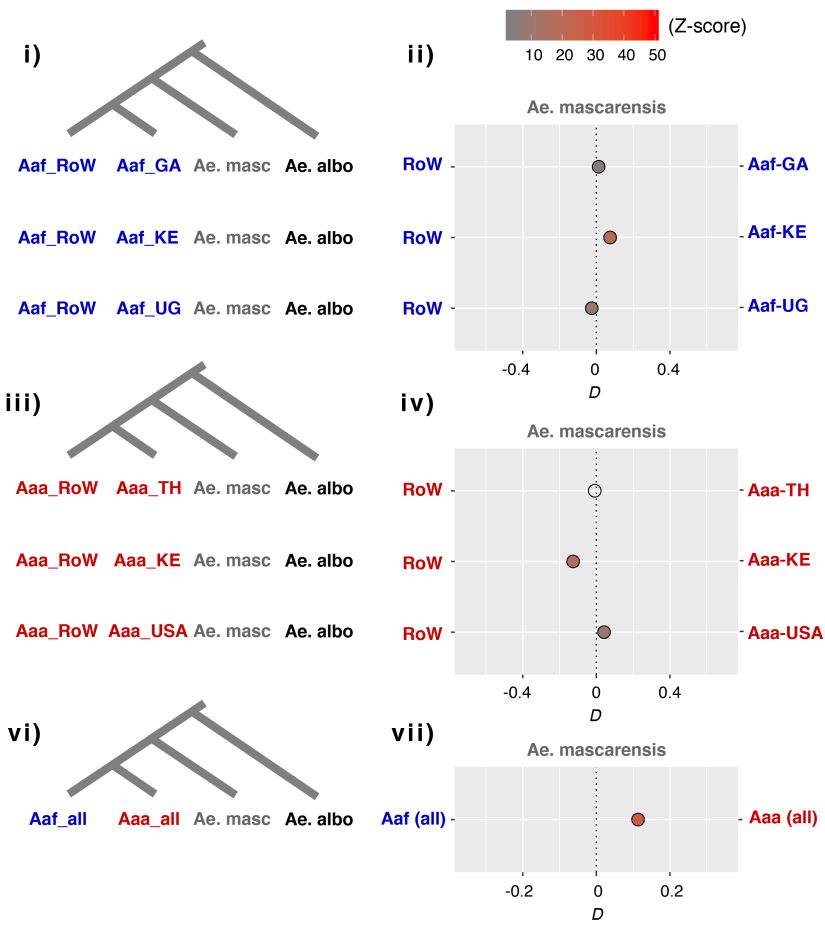

**Fig S5c:**

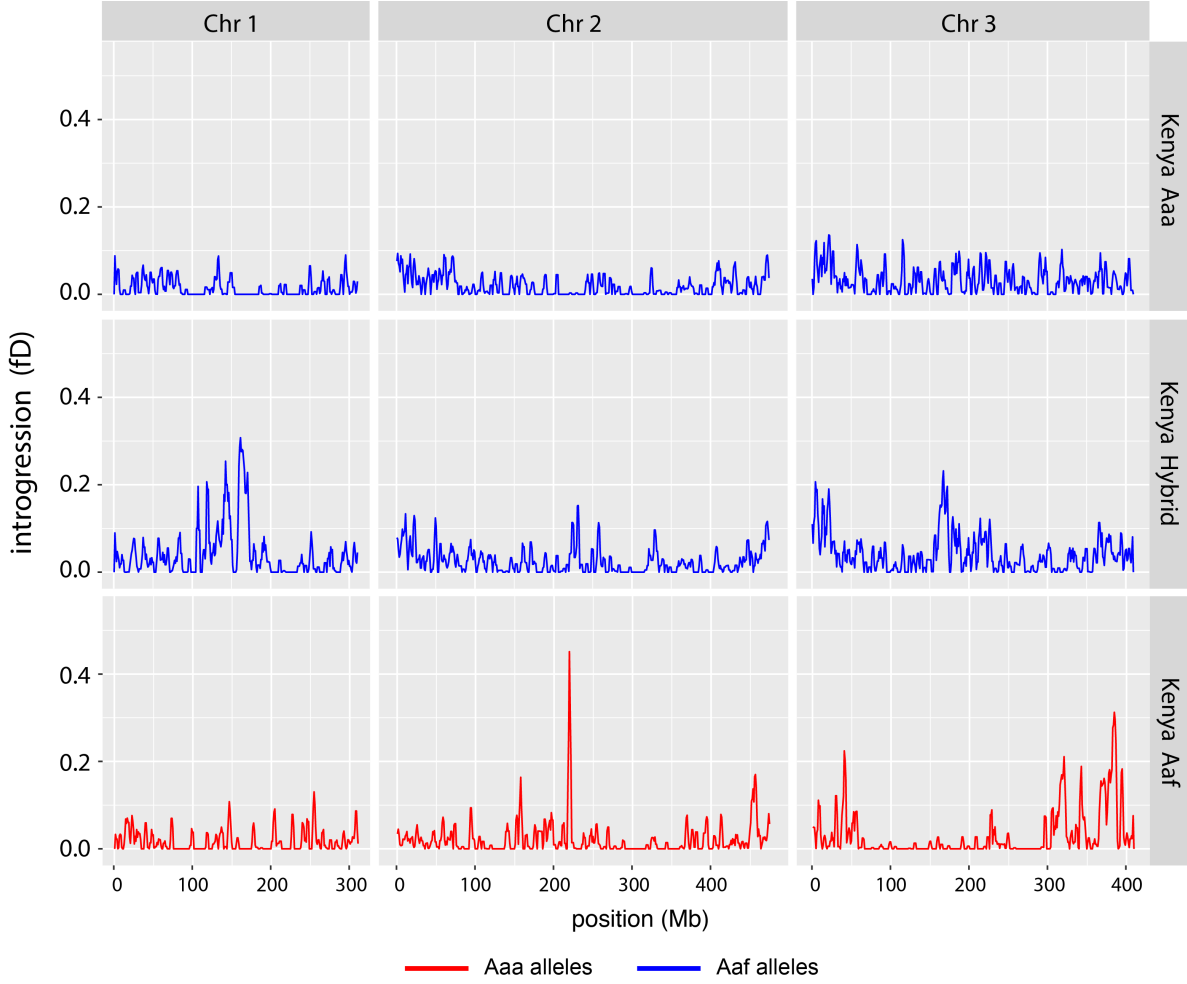

**Fig S5d:**

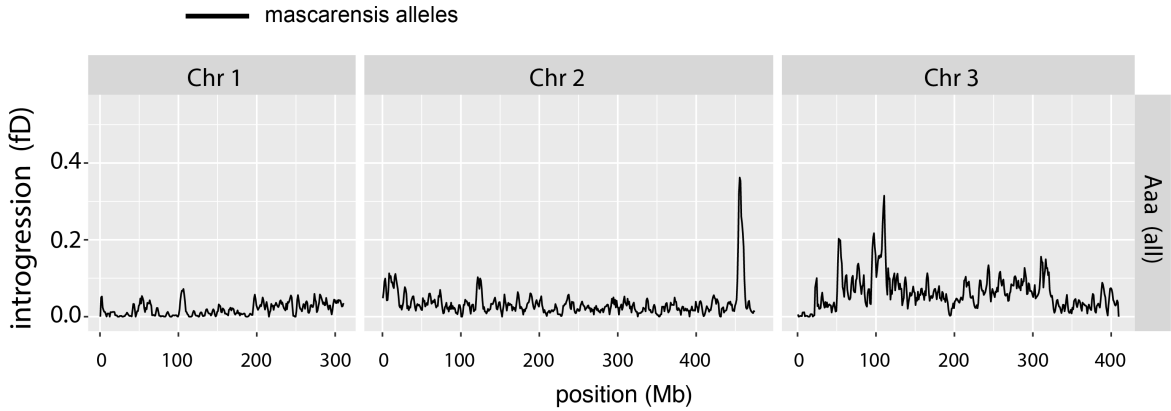

Supplement: Supplementary file 5 — Additional file 5: Figure S5. All Applied Introgression Tests. Patterson’s D was used to assess introgression between a potential introgressing clade and pair of putative sister clades, with an outgroup used to determine derived alleles for the other three clades; 5a) introgression into individual colonies was compared by applying Patterson’s D statistic to each colony as compared to the other two colonies of the same subspecies (e.g we examined Aaf introgression into Kenya_Aaa vs Thai_Aaa + USA_Aaa using Ae. mascarensis as the outgroup). Specific tests applied are given in figures i,iii,v, and the results for each shown in figs ii,iv,vi. Significance was tested by block jackknifing. 5b) The same test was applied to introgression between mascarensis and any individual population (i-iv), as well as between Ae mascarensis and all Aaf or Aaa populations (v-vi); all inter-specific tests used Ae albopictus as the outgroup. 5c) Significant introgression was detected in between Aaf and Kenya_Aaa, between Aaa and Kenya_Aaf, indicating bidirectional introgression between the two subspecies in Kenya. Martin’s fD statistic was applied to identify specific introgressed loci in these two populations and was also applied to testing for Aaf introgression into the ‘hybrid’ population in this region. 5d) significant introgression was also detected between Ae. mascarensis and global Aaa populations. Notably one fD peaks at the distal end of chromosome 2q appeared to have introgressed between Aaa and Kenya_Aaf and Aaa and Ae mascarensis. [file 12915_2020_757_MOESM5_ESM.pdf]

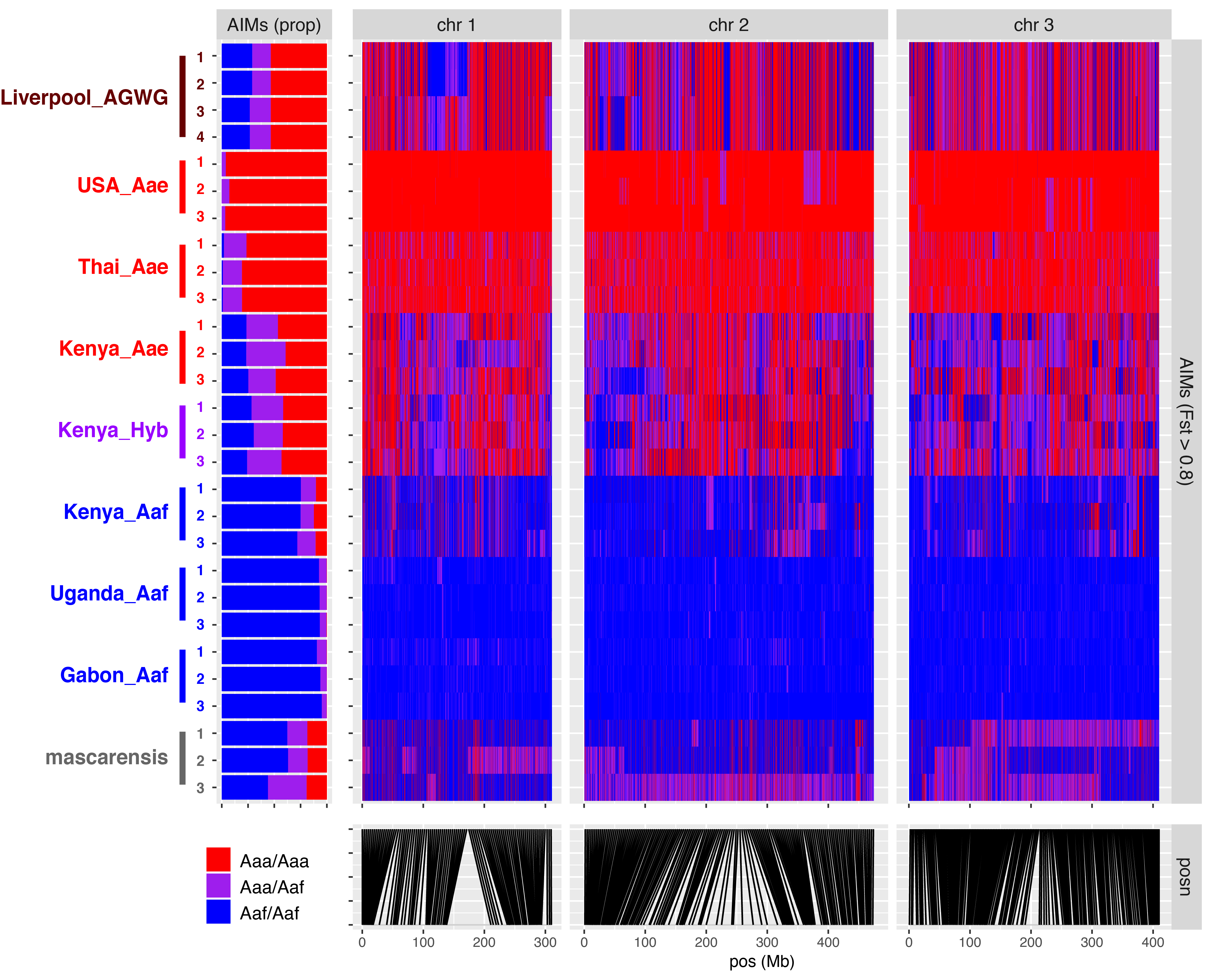

Supplement: Supplementary file 6 — Additional file 6: Figure S6. Aegypti / Formosus Ancestrally-Informative Markers, Liverpool colony. Contrary to expectation, the Ae. aegypti Liverpool strain used for sequencing was not a clear Aaa strain, but instead demonstrated evidence of both Aaa and Aaf alleles. This is consistent with a west African origin and prior evidence from Crawford et al. of forms ancestral to Aae/Aaf in this region [40]. [file 12915_2020_757_MOESM6_ESM.png]

**Fig S7a**

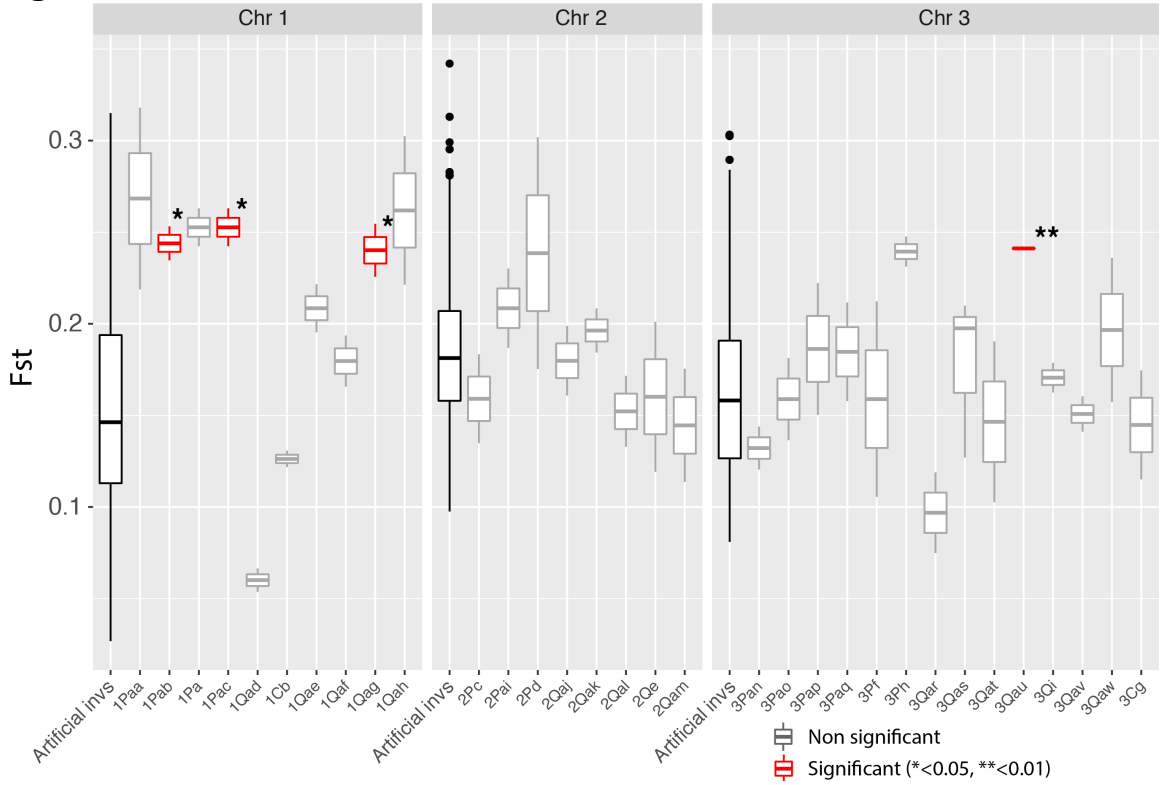

**Fig S7b**

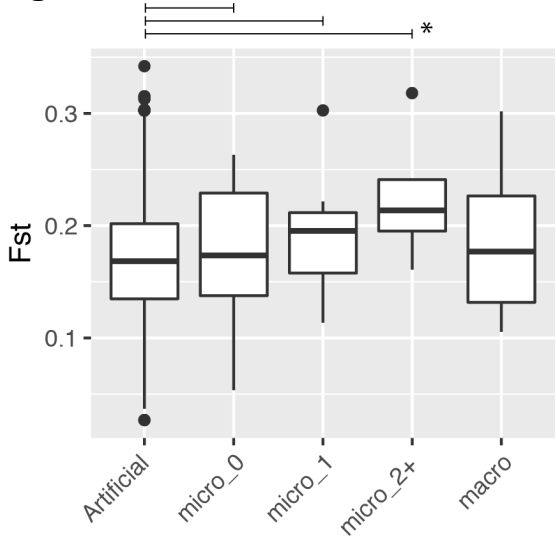

Supplement: Supplementary file 7 — Additional file 7: Figure S7. Elevation of Fst Within Inversions. Fst between pure Aaa and Aaf colonies was calculated in 1mb windows across the genome and windows that contained either an entire microinverisons or a larger inversion breakpoint were compared to a set of artificial inversions constructed of ‘breakpoints’ containing similar levels of TEs and repeats. Fst was compared between categories via Wilcoxon rank-sum test. A) Of the 32 inversions 4 (shown in red) showed significantly elevated levels of Fst indicating a higher degree of differentiation between subspecies; three on chromosome one, the fourth inversion showing elevated Fst is 3Qau, containing 10 genes, 8 of which are odorant binding proteins previously seen to be differentiated between the two subspecies. B) categories of inversions: macroinversions, microinversions without genes, with one gene, and with many genes, were compared to artificial inversions; while polygenic inversions did show elevated Fst compared to artificial inversions, there was no significant difference found when comparing 0 and 1-gene microinversions to polygenic microinversions. [file 12915_2020_757_MOESM7_ESM.pdf]

Fig S8a:

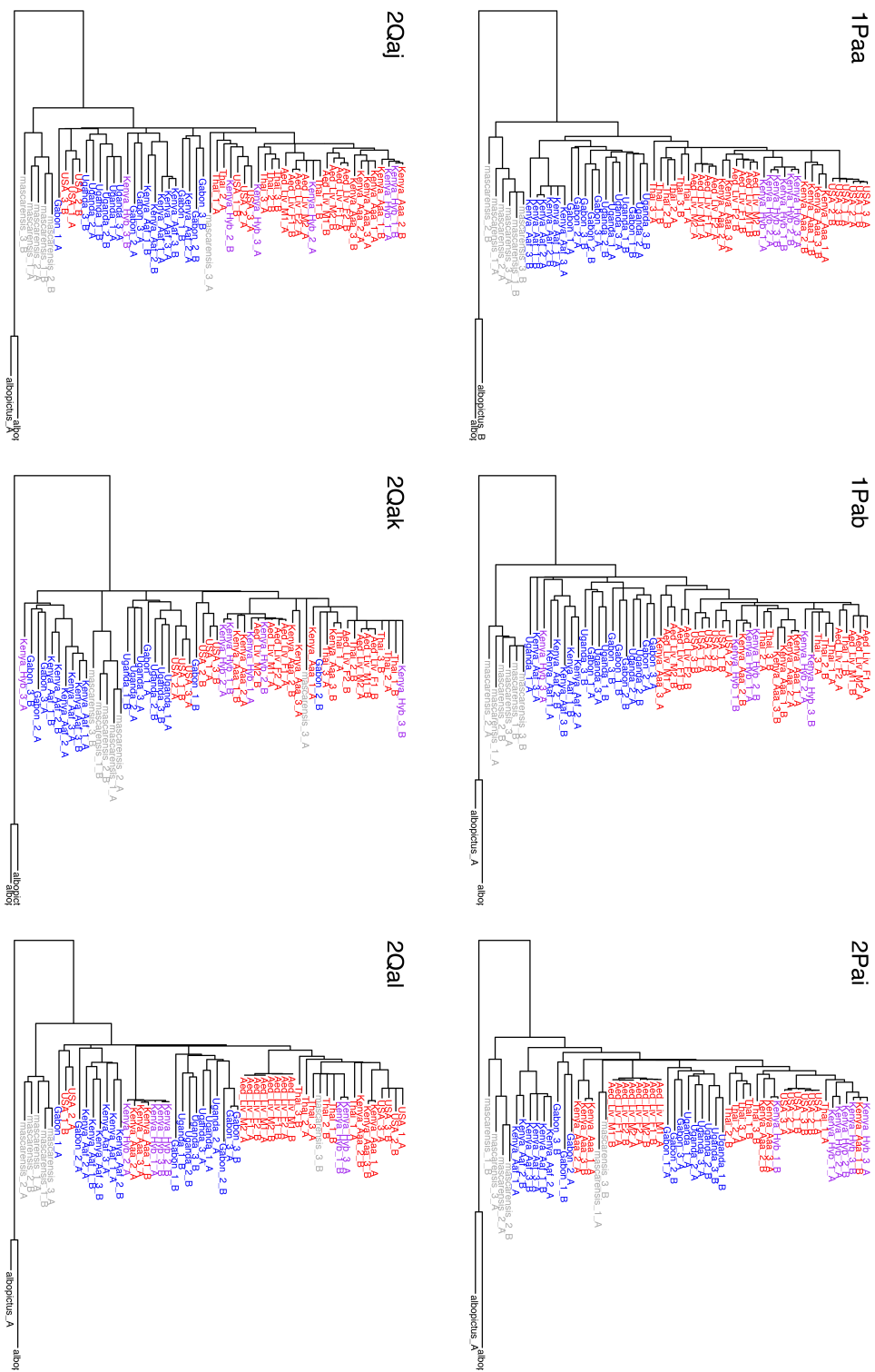

**Fig S8b:**

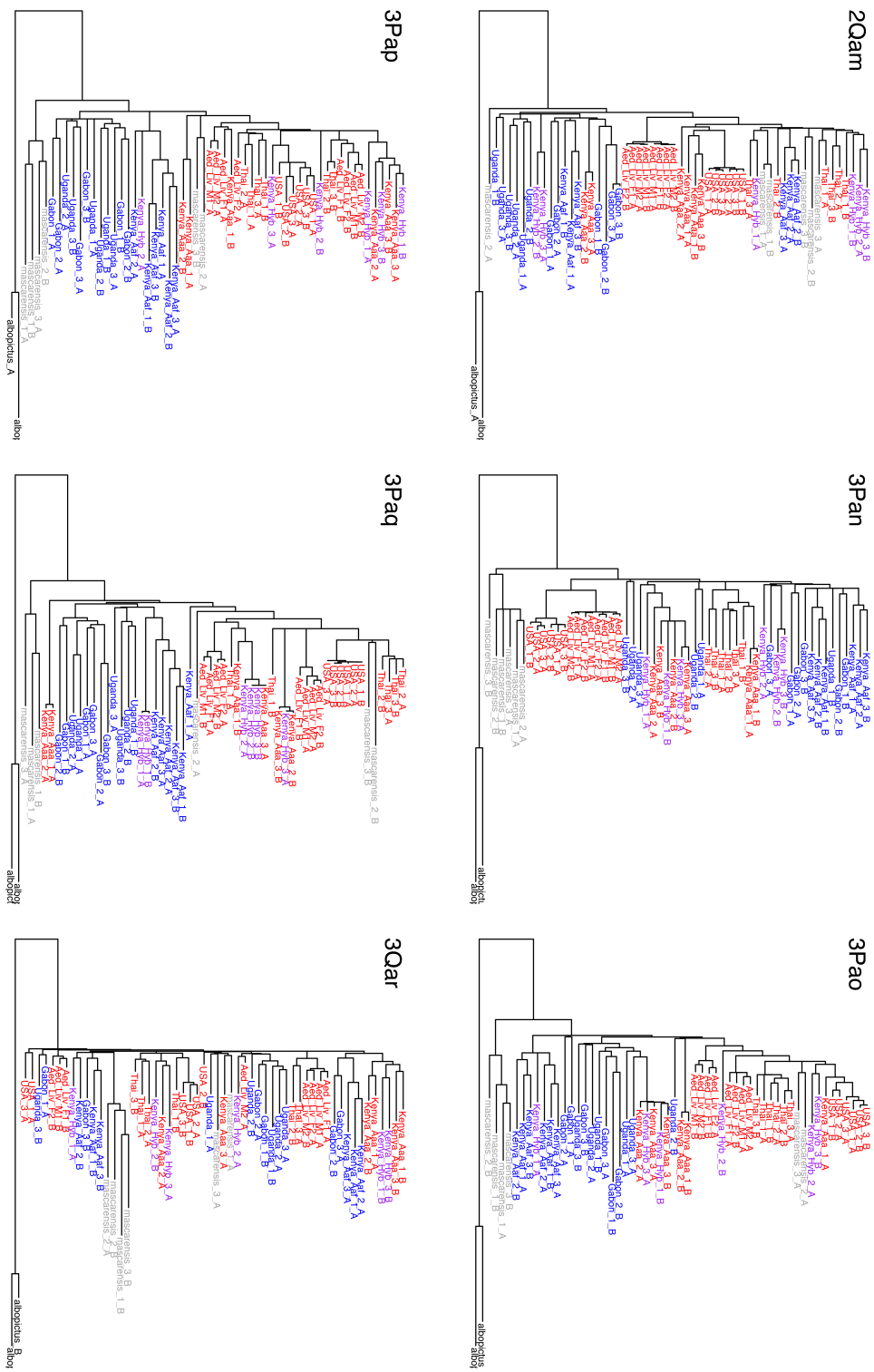

Fig S8c:

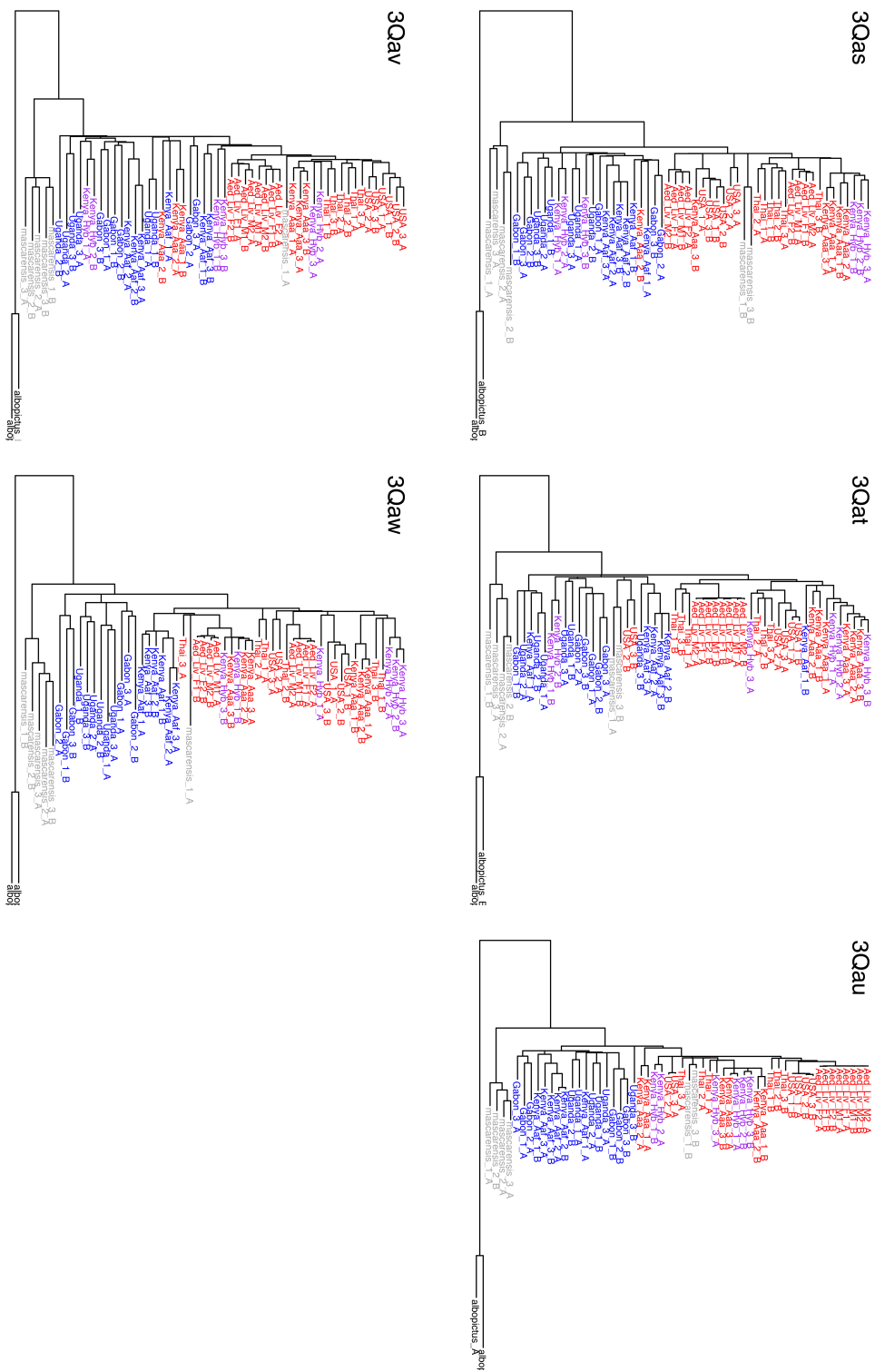

Supplement: Supplementary file 8 — Additional file 8: Figure S8. Within-Inversion Phylogenies. Maximum parsimony phylogenies were derived from the 1 M region surrounding each microinversion in order to establish the unique evolutionary history of these regions. In many cases haplotypes did not cluster into clean sub-species clades, but instead indicated extensive introgression of haplotypes from global Aaa populations into local sylvatic forms. [file 12915_2020_757_MOESM8_ESM.pdf]
