# Supplementary material for: Linked-read sequencing identifies abundant microinversions and introgression in the arboviral vector Aedes aegypti
Source: BMC Biol. 2020 Mar 12;18:26. doi: 10.1186/s12915-020-0757-y (PMC7068900; doi:10.1186/s12915-020-0757-y)
Supplement: Supplementary file 9 — Additional file 9: Table S1. fD elevation in inverted regions. [file 12915_2020_757_MOESM9_ESM.docx]

**Table S1 : fD elevation in inverted regions**

| **Introgression:** | ***fD inversions*** | ***fD uninverted*** | ***P*** |
| --- | --- | --- | --- |
| Aaa <-> Kenya_Aaf | 0.022 | 0.027 | 0.99995 |
| Aaf <-> Kenya_Aaa | 0.022 | 0.024 | 0.88515 |
| Aaa <-> Ae mascarensis. | 0.043 | 0.034 | 0.00025 |
