# Supplementary material for: Linked-read sequencing identifies abundant microinversions and introgression in the arboviral vector Aedes aegypti
Source: BMC Biol. 2020 Mar 12;18:26. doi: 10.1186/s12915-020-0757-y (PMC7068900; doi:10.1186/s12915-020-0757-y)
Supplement: Supplementary file 10 — Additional file 10: Table S2. Microinversion genes. [file 12915_2020_757_MOESM10_ESM.docx]

**Table S2 : Microinversion genes**

| **Inversion** | **position** | **gene_ID** | **Name** | **description** |
| --- | --- | --- | --- | --- |
| 1paa | 1:3662097-3696373 | AAEL023161 | |  |
|  |  | **AAEL026435** | |  |
|  |  | **AAEL023562** | |  |
|  |  | AAEL011198 | mRpL38 | mitochondrial ribosomal protein, L38, putative |
| 1pab | 1:12833450-12864379 | AAEL007657 | | low-density lipoprotein receptor (ldl) |
|  |  | AAEL020425 | |  |
| 1pac | 1:110426504-110463452 |  |  |  |
| 1qad | 1:151766620-151847533 | AAEL025301 | |  |
|  |  | AAEL022861 | |  |
| 1qae | 1:258805045-258838552 | **AAEL005945** | **GPRDOP4** | **GPCR Dopamine Family** |
| 1qaf | 1:261838607-261877216 |  |  |  |
| 1qag | 1:273812946-273859121 |  |  |  |
| 1qah | 1:288864540-288902671 | **AAEL027699*** | |  |
| 2pai | 1:125768796-125807158 | AAEL017560 | |  |
|  |  | AAEL027444 | |  |
| 2qaj | 2:276157193-276249519 | AAEL022937 | |  |
|  |  | **AAEL020943** | |  |
|  |  | **AAEL019634** | |  |
|  |  | **AAEL020352** | |  |
| 2qak | 2:312677653-312836936 | AAEL007271 | | basic helix-loop-helix zip transcription factor |
|  |  | AAEL027808 | |  |
|  |  | **AAEL021441** | |  |
|  |  | **AAEL022367** | |  |
| 2qal | 2:387870950-387930807 | **AAEL018320** | |  |
| 2qam | 2:456306790-456341898 | **AAEL019474** | |  |
| 3pan | 3:43852147-43896847 |  |  |  |
| 3pao | 3:61571542-61611075 | AAEL027940 | |  |
|  |  | AAEL024133 | |  |
| 3pap | 3:72073163-72216512 |  | |  |
| 3paq | 3:83753676-83794150 | **AAEL027400** | |  |
|  |  | AAEL020893 | |  |
| 3qar | 3:198751700-198836215 | AAEL026476 | |  |
| 3qas | 3:213245322-213670971 | **AAEL021082** | |  |
| 3qat | 3:229688967-229782178 |  |  |  |
| 3qau | 3:257058020-257252866 | AAEL002599 | |  |
|  |  | AAEL022118 | |  |
|  |  | **AAEL002598** | OBP15 | odorant binding protein OBP15 |
|  |  | **AAEL002618** | OBP65 | odorant binding protein OBP65 |
|  |  | **AAEL002602** | | DNA photolyase |
|  |  | **AAEL002605** | OBP14 | odorant binding protein OBP14 |
|  |  | **AAEL002591** | OBP13 | odorant binding protein OBP13 |
|  |  | **AAEL026358** | |  |
|  |  | **AAEL026698** | |  |
|  |  | **AAEL002617** | OBP12 | odorant binding protein OBP12 |
|  |  | **AAEL002587** | OBP11 | odorant binding protein OBP11 |
|  |  | **AAEL002606** | OBP35 | odorant binding protein OBP35 |
|  |  | **AAEL025953** | |  |
|  |  | **AAEL002626** | | odorant-binding protein 56e, putative |
|  |  | AAEL002624 | | serine protease |
|  |  | AAEL002585 | | serine protease |
|  |  | AAEL002610 | | serine protease |
| 3qav | 3:374671141-374714093 |  |  |  |
| 3qaw | 3:386773996-386844766 |  |  |  |
|  |  |  |  |  |

*1qah found within intron of gene AAEL027699
