# Supplementary material for: Linked-read sequencing identifies abundant microinversions and introgression in the arboviral vector Aedes aegypti
Source: BMC Biol. 2020 Mar 12;18:26. doi: 10.1186/s12915-020-0757-y (PMC7068900; doi:10.1186/s12915-020-0757-y)
Supplement: Supplementary file 11 — Additional file 11: Table S3. Non-synonymous variants: Inversion 3qau. [file 12915_2020_757_MOESM11_ESM.docx]

**Table S3 : non-synonymous variants: Inversion 3qau**

| **Gene** | **Gene Name** | **SNP** | **Codon** | **Consequence** | **MAF** | | | **Fst** | **Senegal urban / forest [40]** |
| --- | --- | --- | --- | --- | --- | --- | --- | --- | --- |
|  |  |  |  |  | **Aae** | **Aaf** | **Masc** | **Aae/Aaf** |  |
| AAEL002598 | OBP15 | 3:257082112:C/T | G 61 A | Missense / splice_region | 0.00 | 0.00 | 0.33 |  |  |
|  |  | 3:257082153:G/C | C 20 G | missense | 0.00 | 0.00 | 0.67 |  |  |
| **AAEL002618** | **OBP65** | 3:257095024:C/T | G 401 A | missense | 0.00 | 0.00 | 0.17 |  |  |
|  |  | 3:257095093:G/A | C 332 T | missense | 0.00 | 0.25 | 0.00 | 0.20 | -0.04 |
|  |  | 3:257095181:C/T | G 244 A | missense | 0.00 | 0.17 | 0.00 | 0.10 |  |
|  |  | 3:257095417:C/A | G 73 T | missense | 0.00 | 0.17 | 0.00 | 0.10 | 0.19 |
|  |  | 3:257095508:C/A | G 49 T | stop_gained | 0.00 | 0.08 | 0.00 | 0.00 |  |
|  |  | 3:257095532:G/A | C 25 T | missense | 0.00 | 0.17 | 0.67 | 0.10 |  |
|  |  | 3:257095544:T/C | A 13 G | missense | 0.58 | 0.58 | 1.00 | -0.06 |  |
| AAEL002602 | - | 3:257101533:G/A | G 67 A | missense | 0.00 | 0.00 | 0.00 |  |  |
|  |  | 3:257101624:G/A | G 158 A | missense | 0.00 | 0.00 | 0.00 |  | 0.13 |
|  |  | 3:257101710:A/C | A 244 C | missense | 0.33 | 0.00 | 0.00 | 0.25 |  |
| AAEL002605 | OBP14 | 3:257114222:T/C | A 323 G | missense | 0.00 | 0.00 | 0.50 |  |  |
|  |  | 3:257114244:C/T | G 301 A | missense | 0.00 | 0.00 | 0.00 |  |  |
|  |  | 3:257114288:G/A | C 257 T | missense | 0.00 | 0.33 | 0.00 | 0.30 | -0.04 |
|  |  | 3:257114319:C/T | G 226 A | missense | 0.42 | 0.00 | 0.33 | 0.40 | 0.16 |
|  |  | 3:257114364:T/G | A 181 C | missense | 0.00 | 0.00 | 0.67 |  |  |
|  |  | 3:257114561:A/G | T 41 C | missense | 0.00 | 0.00 | 0.67 |  |  |
|  |  | 3:257114571:C/T | G 31 A | missense | 0.00 | 0.17 | 0.00 | 0.10 | 0.07 |
|  |  | 3:257114579:A/G | T 23 C | missense | 0.58 | 0.00 | 0.33 | 0.54 | 0.08 |
|  |  | 3:257114589:C/G | G 13 C | missense | 0.00 | 0.17 | 0.00 | 0.10 | 0.07 |
|  |  | 3:257114592:T/A | A 10 T | missense | 0.00 | 0.00 | 0.67 |  |  |
| AAEL002591 | OBP13 | 3:257121379:A/T | A 10 T | missense | 0.58 | 0.00 | 0.33 | 0.54 | 0.08 |
|  |  | 3:257121409:G/T | G 40 T | missense | 0.00 | 0.17 | 0.00 | 0.10 | 0.13 |
|  |  | 3:257121420:G/T | G 51 T | Missense / splice_region | 0.00 | 0.00 | 0.00 |  |  |
|  |  | 3:257121499:A/T | A 71 T | missense | 0.00 | 0.08 | 0.00 | 0.00 |  |
|  |  | 3:257121609:A/C | A 181 C | missense | 0.00 | 0.00 | 0.50 |  |  |
|  |  | 3:257121691:A/C | A 263 C | missense | 0.00 | 0.25 | 0.00 | 0.20 |  |
| AAEL002617 | OBP12 | 3:257142884:C/T | G 247 A | missense | 0.00 | 0.00 | 0.50 |  |  |
|  |  | 3:257143045:C/T | G 86 A | missense | 0.58 | 0.00 | 0.33 | 0.54 | 0.08 |
|  |  | 3:257143174:A/C | T 16 G | missense | 0.00 | 0.00 | 0.00 |  | 0.07 |
| **AAEL002587** | **OBP11** | 3:257150622:A/T | A 12 T | missense | 0.00 | 0.00 | 0.00 |  |  |
|  |  | 3:257150627:C/A | C 17 A | stop_gained | 0.00 | 0.08 | 0.00 | 0.00 | 0.01 |
|  |  | 3:257150653:G/C | G 43 C | missense | 0.00 | 0.25 | 0.00 | 0.20 | 0.15 |
|  |  | 3:257151007:C/T | C 256 T | missense | 0.00 | 0.00 | 0.50 |  |  |
|  |  | 3:257151017:A/G | A 266 G | missense | 0.00 | 0.08 | 0.00 | 0.00 |  |
|  |  | 3:257151025:G/A | G 274 A | missense | 0.00 | 0.00 | 0.00 |  |  |
|  |  | 3:257151029:C/A | C 278 A | missense | 0.00 | 0.00 | 0.17 |  |  |
|  |  | 3:257151103:G/C | G 352 C | missense | 0.00 | 0.00 | 0.67 |  | 0.09 |
|  |  | 3:257151104:C/T | C 353 T | missense | 0.00 | 0.17 | 0.00 | 0.10 |  |
| AAEL002606 | OBP35 | 3:257164890:C/T | G 40 A | missense | 0.33 | 0.00 | 0.00 | 0.25 |  |
|  |  | 3:257164907:G/A | C 23 T | missense | 0.00 | 0.17 | 0.00 | 0.10 |  |
| AAEL002626 | OBP56e | 3:257205937:T/A | T 2 A | start_lost | 0.00 | 0.00 | 0.17 |  |  |
|  |  | 3:257205954:T/A | T 19 A | missense | 0.00 | 0.08 | 0.00 | 0.00 |  |
|  |  | 3:257205958:G/A | G 23 A | missense | 0.00 | 0.00 | 0.67 |  |  |
|  |  | 3:257206023:C/T | C 88 T | missense | 0.67 | 0.92 | 1.00 | 0.08 | 0.08 |
|  |  | 3:257206038:C/T | C 103 T | stop_gained | 0.00 | 0.17 | 0.00 | 0.10 | 0.00 |
|  |  | 3:257206050:T/C | T 115 C | missense | 0.00 | 0.00 | 0.50 |  |  |
|  |  | 3:257206051:C/T | C 116 T | missense | 0.00 | 0.00 | 0.00 |  |  |
|  |  | 3:257206072:A/T | A 137 T | missense | 0.00 | 0.08 | 0.00 | 0.00 |  |
|  |  | 3:257206312:C/T | C 260 T | missense | 0.00 | 0.08 | 0.00 | 0.00 |  |
|  |  | 3:257206601:G/A | G 481 A | missense | 0.00 | 0.17 | 0.00 | 0.10 | 0.07 |
